# Does Biofunctionalization Make ZnO and TiO_2_ Nanoparticles Safer? A Critical Review of Their Changing Toxicological Identity

**DOI:** 10.3390/toxics14100904

**Published:** 2026-10-12

**Authors:** Liliana Cepoi, Tatiana Chiriac, Ludmila Rudi, Lilit Gabrielyan, Anna Karapetyan

**Affiliations:** 1 Institute of Microbiology and Biotechnology, Technical University of Moldova, MD-2028 Chisinau, Moldova; tatiana.chiriac@imb.utm.md (T.C.); ludmila.rudi@imb.utm.md (L.R.); 2 Department of Biochemistry, Microbiology and Biotechnology, Biology Faculty, Yerevan State University, Yerevan 0025, Armenia; lgabrielyan@ysu.am; 3 Research Institute of Biology, Yerevan State University, Yerevan 0025, Armenia; annakarapetyan@ysu.am; 4 Department of Human and Animal Morphology and Physiology, Biology Faculty, Yerevan State University, Yerevan 0025, Armenia

**Keywords:** ZnO NPs, TiO_2_ NPs, nano–bio interface, surface modification, protein corona, dissolution, biological transformation, safe-by-design

## Abstract

ZnO and TiO_2_ nanoparticles are increasingly biofunctionalized to improve stability, biological recognition, targeting, and compatibility. However, whether biofunctionalization inherently makes these nanoparticles safer remains unresolved. Rather than cataloguing biofunctionalization strategies, this critical review evaluates whether toxicological changes can be attributed specifically to deliberate biomolecular surface modification and whether any resulting safety advantage persists during exposure-relevant transformations. A structured literature search identified 60 publications for critical synthesis, including 10 comparative studies providing direct evidence on toxicological changes attributable to deliberate biofunctionalization. The available evidence does not support a uniform toxicity-reducing effect. Depending on the nanoparticle-biointerface combination and exposure context, biofunctionalization can reduce toxicity, produce coating-dependent changes, increase biological accessibility, or redirect reactivity toward selected biological targets. These outcomes arise from changes in surface charge, aggregation, colloidal stability, cellular interactions, and biointerface evolution and are material-specific: ZnO additionally requires consideration of dissolution and Zn^2+^ release, whereas TiO_2_ behavior is more strongly influenced by particulate persistence and surface-mediated reactivity. We therefore conceptualize toxicological identity as an evolving, exposure-dependent profile rather than an intrinsic material property. Within a safe-by-design framework, biofunctionalization is advantageous only when it reduces non-target toxicity, preserves or improves the intended function, and maintains this advantage after exposure-relevant transformations.

## 1. Introduction

Zinc oxide nanoparticles (ZnO NPs) and titanium dioxide nanoparticles (TiO_2_ NPs) are among the most widely applied oxide nanomaterials due to their combination of optical, photocatalytic, antimicrobial, and semiconducting properties, together with the possibility of tailoring their size, morphology, and surface chemistry. ZnO NPs are used or investigated in antimicrobial formulations, delivery systems, UV-protective products, and various biomedical and food-related applications, whereas TiO_2_ NPs are of particular interest for photoprotection, photocatalysis, antimicrobial materials, and biomedical systems [1,2,3,4,5]. This versatility has stimulated the development of increasingly sophisticated synthesis and surface-modification strategies, including the use of biological extracts, proteins, peptides, polysaccharides, and biosurfactants to obtain nanoformulations with tunable biological properties [6,7,8].

However, the same properties that confer desirable functionality to ZnO NPs and TiO_2_ NPs may also contribute to their adverse effects. ZnO nanoparticle toxicity has been associated with reactive oxygen species (ROS) generation, oxidative stress, disruption of cellular homeostasis, particle dissolution, and the release of Zn^2+^ ions. For TiO_2_ NPs, surface reactivity and photocatalytic activity, crystalline properties, and ROS generation are important determinants of their biological interactions [1,9,10]. Nevertheless, clearly distinguishing toxicity attributable to the “particle” itself from toxicity arising from products generated during its transformation is difficult. In the case of ZnO NPs, comparisons between nanoparticle exposure and exposure to ionic Zn have demonstrated that dissolution contributes substantially to biological effects. However, it does not fully account for the metabolic response induced by the nanoparticulate form [11]. Thus, the toxicity of a nanomaterial cannot be inferred solely from its nominal chemical composition.

Particle size and shape, specific surface area, crystalline structure, surface charge and chemistry, aggregation state, dissolution rate, and photochemical reactivity can simultaneously influence nanoparticle interactions with biological environments. The surface is particularly important because it constitutes the first point of contact between the inorganic material and the molecules, membranes, and cells in the surrounding environment. Surface modification can alter colloidal stability and the accessibility of reactive surface sites, as well as protein adsorption, cellular recognition, nanoparticle uptake, and biological localization [9,12,13]. Consequently, nanoparticles with the same ZnO or TiO_2_ core may exhibit substantially different biological behaviors when their surfaces differ in composition and functionalization.

In this context, biofunctionalization has emerged as an attractive strategy for controlling the interface between nanomaterials and biological environments. Proteins, peptides, polysaccharides, lipids, biosurfactants, and other biomolecules can modify dispersibility, surface charge, stability, molecular recognition, and interactions with biological targets [6,7]. However, biogenic synthesis, residual biomolecular coatings, and deliberate biofunctionalization are not equivalent processes and do not provide the same level of evidence regarding the contribution of the surface to toxicity [2,3,4,8].

The association of biomolecules with nanoparticle surfaces does not necessarily reduce toxicity. Available comparative evidence indicates that biofunctionalization may attenuate certain adverse effects but may also, under other conditions, increase bioavailability or cellular interactions, preserve the reactivity of the inorganic core, or redirect biological effects toward specific targets [14,15,16,17,18,19,20]. Moreover, the initially engineered surface identity is not necessarily preserved once nanoparticles enter a biological environment. Biomolecular corona formation, aggregation, dissolution, and other environment-dependent transformations can remodel the biointerface and, consequently, the nanomaterial’s biological and toxicological profile [12,13,21].

The present review critically examines whether, and under what conditions, biofunctionalization can make ZnO and TiO_2_ nanoparticles safer. While previous reviews have extensively addressed the synthesis, functionalization, biological applications, and toxicity of metal oxide nanoparticles, the present analysis is specifically organized around the toxicological consequences attributable to deliberate biomolecular surface modification. Particular emphasis is placed on comparative studies that distinguish effects of the inorganic core from those associated with the engineered biointerface, while biogenic synthesis, residual biomolecular coatings, and acquired coronas are treated as conceptually distinct phenomena. Rather than considering biofunctionalization as a fixed material property, we follow the engineered interface through exposure-dependent transformations to determine whether an initial toxicological advantage is preserved, lost, amplified, or redirected. The comparison of ZnO and TiO_2_ further accounts for their distinct transformation pathways, particularly the importance of dissolution and Zn^2+^ release for ZnO versus the greater particulate persistence and surface-mediated reactivity of TiO_2_. Thus, the distinctive contribution of this review is not a catalogue of biofunctionalization strategies, but a critical assessment of whether deliberate biomolecular surface modification produces a demonstrable and persistent toxicological advantage. The central hypothesis of this review is that biofunctionalization should not be regarded a priori as a toxicity-reduction strategy but rather as an intervention at the biointerface that can reconfigure the toxicological identity of ZnO and TiO_2_ nanoparticles.

## 2. Literature Search and Source Selection Strategy

This review used a structured search of the Scopus and Web of Science Core Collection databases to identify publications on the biofunctionalization of ZnO NPs and TiO_2_ NPs, biomolecular surface modification, and the physicochemical, biological, and toxicological consequences of these modifications. The primary search targeted English-language publications from 2020 to 2026. We subsequently included earlier studies only when we identified them as foundational or mechanistically relevant by examining the reference lists of the selected publications.

In Scopus, the search targeting the review’s conceptual core retrieved 315 original research articles and 78 review articles. In contrast, a complementary search addressing food-related contexts, ingestion, and nanoparticle interactions with the gastrointestinal tract identified 81 additional original research articles, yielding a total of 474 records. The complementary Web of Science search identified 367 articles and reviews. We merged and deduplicated the resulting 841 records using DOI and, when a DOI was unavailable, normalized article titles. After removal of 252 duplicates, 589 unique publications remained for screening. Records were excluded during screening or detailed relevance assessment when they did not address ZnO or TiO_2_ nanoparticles, did not involve a biologically relevant surface modification or transformation, provided insufficient information to evaluate the nanoparticle–biointerface relationship, or did not contribute directly to the mechanistic, biological, toxicological, or exposure-related questions addressed in this review. Studies based exclusively on material synthesis or characterization without relevant biological or mechanistic endpoints were also excluded.

**Figure 1:**
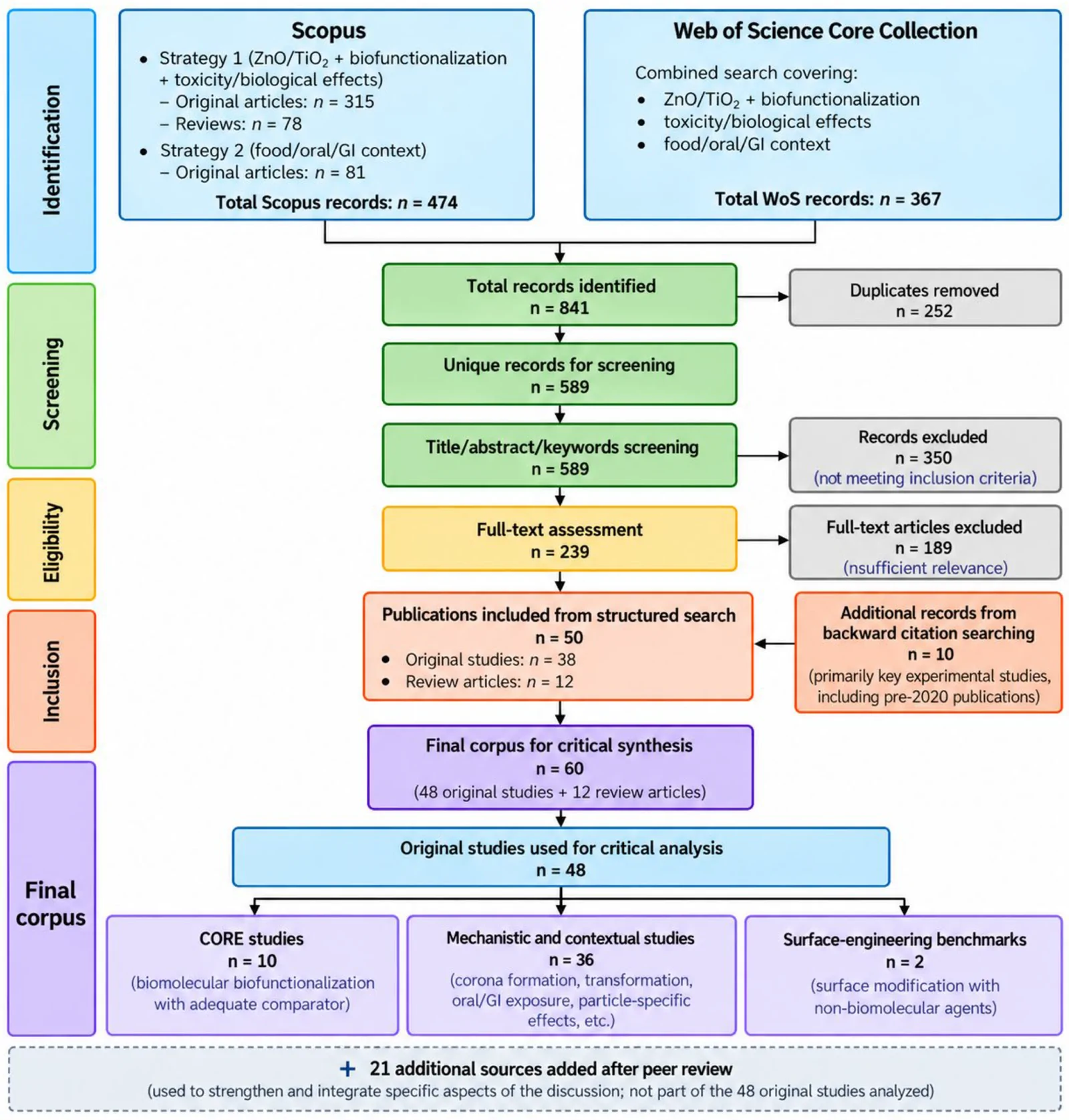
Literature search, study selection, and evidence structure for the critical review.

We prioritized studies involving deliberate surface modification of ZnO NPs or TiO_2_ NPs with different biomolecules, as well as mechanistic studies relevant to biomolecular corona formation, aggregation, dissolution, cellular uptake, gastrointestinal transformations, and oral exposure. Green/biogenic synthesis was not automatically considered equivalent to biofunctionalization; we treated such studies as evidence of biofunctionalization only when experimental evidence supported the presence and functional role of the biomolecular component at the nanoparticle surface. Following screening, 239 publications underwent detailed assessment, of which 189 were excluded. The structured search therefore yielded 50 publications (38 original research articles and 12 reviews). Backward citation searching of the 12 review articles identified an additional 10 experimental studies, resulting in a final corpus of 60 publications. Following peer review, 21 additional references were incorporated to strengthen specific methodological, mechanistic, and contextual aspects of the discussion requested by the reviewers. These supplementary references are indicated separately in Figure 1 and were not included in the original evidence corpus or used to modify the CORE comparative dataset.

For the critical synthesis, we distinguished between direct comparative evidence and mechanistic/contextual studies. The CORE category included only studies involving deliberate biomolecular biofunctionalization of ZnO NPs or TiO_2_ NPs that also incorporated an appropriate comparator, allowing biological or toxicological changes to be attributed to the biofunctionalized surface; 10 studies met these criteria. We also retained two studies involving synthetic surface modification as surface-engineering benchmarks. Importantly, studies that did not meet the CORE criteria were not necessarily excluded from the critical synthesis. Several experimentally relevant studies were retained as supporting evidence when their design did not allow the toxicological effect of deliberate biofunctionalization to be isolated. These included biogenic ZnO systems in which biomolecules participated simultaneously in nanoparticle formation and surface stabilization, multicomponent systems in which biofunctionalization was accompanied by changes in particle size, morphology, defect structure, or additional active components, and studies lacking a matched non-biofunctionalized nanoparticle comparator. Representative examples include the *Arthrospira platensis*-mediated ZnO NPs of El-Belely et al. (2021) [22], in which biomolecules participate in nanoparticle formation during green synthesis; the one-pot curcumin–chitosan–ZnO system of Karthikeyan et al. (2020) [23], which lacks a matched non-biofunctionalized ZnO comparator; and the biofunctionalized defect-engineered ZnO quantum dots of Sarkar et al. (2022) [24], in which surface modification is coupled with changes in defect structure and other material properties. These studies were therefore retained as supporting evidence but not classified as CORE because the specific contribution of biofunctionalization to the observed biological effects could not be isolated.

Accordingly, the evidence was interpreted according to the strength of causal inference it provided. Direct conclusions regarding the effects of biofunctionalization were based primarily on the 10 CORE studies, whereas the remaining original studies provided mechanistic or contextual support. For this reason, not all 48 original studies are discussed individually in the comparative sections; non-CORE studies are cited selectively where they provide mechanistic, methodological, or contextual evidence relevant to the interpretation of biofunctionalization. Given the limited number of CORE studies and the unequal comparative evidence available for ZnO and TiO_2_, material-specific conclusions were interpreted cautiously and were not extrapolated from one oxide to the other without supporting evidence. Broader principles concerning dynamic biointerface remodeling, exposure-dependent transformations, and the need for appropriate comparators were considered cross-cutting concepts when supported by convergent mechanistic evidence. This approach was designed to provide a mechanistically oriented critical synthesis rather than a systematic review or meta-analysis.

## 3. Biofunctionalization: Definition and Conceptual Boundaries

The terms surface modification, surface functionalization, biofunctionalization, biogenic synthesis, green synthesis, biomolecular coating, and biomolecular corona are frequently used in the nanoparticle literature, sometimes with partially overlapping meanings. In the context of the present review, which focuses on the relationship between surface properties and toxicity, this ambiguity is more than merely terminological because how biofunctionalization is defined determines which changes in nanoparticle biological behavior can be causally attributed to the biological surface layer. Broadly, surface functionalization refers to introducing chemical groups, ligands, or coatings that impart new properties to nanoparticles. It may involve synthetic or biological modifiers and can be achieved through physical adsorption, electrostatic interactions, coordination, covalent binding, or linker molecules [6]. In this review, however, we use biofunctionalization in a more restrictive sense: the intentional modification of a nanoparticle interface through association of one or more biomolecules, whereby they become functional components of the nanoparticle surface.

This definition encompasses proteins, peptides, polysaccharides, lipids, biosurfactants, and other biological molecules deliberately attached to or adsorbed onto ZnO NPs or TiO_2_ NPs. Importantly, biomolecules must not merely be present in the system; they must contribute to the formation and properties of the nanoparticle-environment interface. Depending on their nature and mode of association, the biological layer may act as a colloidal stabilizer, recognition ligand, mediator of membrane interactions, or barrier between the oxide surface and the surrounding environment. For example, antimicrobial peptides immobilized on TiO_2_ NPs remain functional components of the interface and can modulate particle interactions with membranes of different compositions [25]. Similarly, proteins such as albumin can be used as functional coatings to modify the colloidal stability and cellular interactions of TiO_2_ NPs [15,26]. Biosurfactants represent a particular form of biofunctionalization because their amphiphilic structure enables their association with ZnO or TiO_2_ surfaces, thereby modifying the nanoparticle-environment interface and, consequently, colloidal behavior and biological interactions [7,20,27].

An essential distinction must be made between biofunctionalization and biogenic synthesis. In biologically mediated synthesis, plant extracts, microorganisms, algae, cyanobacteria, or their metabolic products may participate in nanoparticle formation while simultaneously performing complexing, stabilizing, and capping functions [3,4,8]. In such systems, the organic surface layer often results from the synthesis process rather than a distinct functionalization step. Moreover, biological extracts are complex mixtures, and researchers do not always identify or quantify the biomolecules responsible for particle formation and stabilization. The literature on biogenic ZnO NPs acknowledges this limitation: the biochemical composition of the reaction media may be incompletely characterized, and its variability can influence the properties of the final product [4].

For this reason, in the present review, the terms biogenic or green-synthesized are not automatically considered synonymous with biofunctionalized. An illustrative example is provided by ZnO NPs synthesized using *Arthrospira platensis*, in which proteins, polysaccharides, and enzymes from the cyanobacterial filtrate explicitly participate in nanoparticle formation, stabilization, and capping [22]. Such material undoubtedly contains a biological component associated with its formation process. However, if nanoparticles produced through this process differ from chemically synthesized ZnO NPs in size, morphology, crystalline defects, aggregation state, and surface composition, the observed biological effect cannot be attributed exclusively to surface-associated biomolecules. The same issue arises in other ZnO NPs systems synthesized using plant extracts, fungal proteins, or microbial exopolysaccharides [28,29,30]. These studies help explain the biointerface. Still, they represent a different category of evidence from experiments in which the same nanoparticles are compared before and after introducing a defined biomolecular layer.

At the other end of the spectrum lies synthetic surface modification. Synthetic polymers, conventional surfactants, and organic ligands can markedly alter nanoparticle stability, protein adsorption, cellular uptake, and toxicity, but they do not constitute biofunctionalization as defined in this review. Accordingly, studies in which ZnO NPs were modified with polyethylene glycol (PEG), aminopropyltriethoxysilane (APTES), cetyltrimethylammonium bromide (CTAB), or sodium dodecyl sulfate (SDS) were not included in the CORE evidence base, even when their comparative experimental designs were highly informative [31,32]. Instead, these studies are treated as surface-engineering benchmarks because they clearly demonstrate that surface modification can alter biological responses without changing the nominal chemical identity of the nanoparticle core. They therefore provide a mechanistic reference framework for interpreting biofunctionalization without artificially broadening its definition.

A second important conceptual distinction concerns the difference between deliberate biofunctionalization and the formation of an acquired biomolecular corona. In the former case, biomolecules were intentionally introduced before biological exposure and constitute part of the initial nanoparticle design. In the latter, biomolecules adsorb spontaneously after nanoparticles contact a biological or environmental medium. The corona may contain not only proteins but also lipids, metabolites, polysaccharides, and components of organic matter, and its composition depends on both nanoparticle properties and the composition of the exposure medium [12,13]. The literature on the eco-corona explicitly highlights that proteins, exopolymers, metabolites, and dissolved organic matter can all contribute to forming this acquired interface.

## 4. Biofunctionalization Strategies for ZnO and TiO_2_ Nanoparticles

The strategies used to biofunctionalize ZnO and TiO_2_ nanoparticles can be examined according to the nature of the biomolecule, its mode of association with the surface, and the degree of control over the resulting interface. The literature included in this review reveals considerable diversity, ranging from relatively simple adsorption of proteins and peptides to oriented conjugation through linker molecules and the formation of hybrid structures involving biopolymers or biosurfactants. These approaches are not equivalent: they differ in association stability, the density and orientation of surface-bound biomolecules, the accessibility of the inorganic surface, and the extent to which the interface’s biological function can be controlled.

### 4.1. Biofunctionalization of ZnO NPs and TiO_2_ NPs with Proteins and Peptides

Within the analyzed corpus, evidence for deliberate biofunctionalization with proteins and peptides is more extensive for TiO_2_ NPs than for ZnO NPs. This asymmetry is important because it reflects differences in the maturity of the available experimental evidence and limits direct comparisons between the two nanoparticle types.

A relatively simple strategy uses food proteins to modify the TiO_2_ NPs surface. Vian et al. precoated nanoparticles with gelatin or β-lactoglobulin, producing dispersions with properties distinct from those of uncoated TiO_2_ [18]. This type of biofunctionalization matters because the biomolecule primarily modifies the interface and colloidal behavior, without introducing a specific biological targeting mechanism. In other systems, bovine serum albumin (BSA) is used as a protein coating or as a component of more complex constructs, including TiO_2_-BSA systems subsequently functionalized with additional components [15,26]. Such architectures demonstrate that a protein can serve both as a direct surface modifier and as an intermediate platform for further functionalization.

Peptides allow finer control over interfacial properties because their sequence, charge, hydrophobicity, and recognition motifs can be tailored. Studies on TiO_2_ nanoparticles functionalized with antimicrobial peptides are particularly illustrative [22,33,34]. Adsorption of the cationic peptide LL-37 onto TiO_2_ is concentration-dependent and results in a pronounced reversal of surface charge; at sufficiently high surface coverage, the nanoparticles acquire a net positive ζ-potential and increased colloidal stability [34]. In this case, biofunctionalization does not require a complex covalent architecture: peptide adsorption is sufficiently stable to generate a functional interface and maintain its characteristics over the course of the experiments.

More importantly, from a surface-design perspective, peptide identity can be rationally modified. Comparison of KYE21 with its more hydrophobic variant WWWKYE21 showed that introducing a tryptophan-rich segment increases peptide association with TiO_2_ and subsequently influences interactions between the functionalized nanoparticles and bacterial membranes [31]. Related studies using LL-37 variants similarly demonstrated that modifying peptide hydrophobicity can alter their binding efficiency to TiO_2_ [32]. Peptides should therefore not be regarded merely as a “biological coating” but rather as tunable molecular elements of surface design.

An additional level of control is provided by receptor-targeted biofunctionalization. Dayan et al. (2018) used dihydrolipoamide dehydrogenase (DLDH) modified with an arginine–glycine–aspartic acid (RGD) sequence to construct the TiO_2_-DLDH-RGD complex, in which the protein mediates association with TiO_2_, while the RGD motif confers integrin-recognition capability [19]. This strategy illustrates the distinction between simple biomolecular coating and targeted biofunctionalization, in which the surface architecture is deliberately designed to introduce a defined recognition function.

### 4.2. Biofunctionalization with Polysaccharides and Polymeric Matrices

Karthikeyan prepared ZnO, chitosan-ZnO (CZnO), and alginate-ZnO (AZnO) through a one-pot process, enabling the formation of organic-inorganic hybrid structures and comparison of the effects associated with the two biopolymers [17]. However, this example also highlights an important methodological consideration: in one-pot strategies, nanoparticle formation and biopolymer association occur simultaneously. Consequently, the biopolymer may influence not only the final product’s surface but also particle nucleation, growth, and organization. Such materials are therefore better described as biofunctionalized hybrid nanostructures rather than preformed nanoparticles subsequently coated. Accordingly, attribution of the observed effects exclusively to surface modification requires caution.

Greater complexity can be achieved by combining multiple biomolecular components. Chitosan–curcumin ZnO NP systems (CCZ), for example, have been constructed as layered or hybrid structures in which the biopolymer and bioactive compound are integrated with the ZnO component [23]. Microscopic and spectroscopic characterization demonstrated the presence of the organic components and interactions among curcumin, chitosan, and ZnO NPs. However, as architecture becomes more complex, attributing a biological change to a single surface component becomes increasingly difficult. This issue is particularly relevant to subsequent toxicological interpretation. It explains why multicomponent hybrid materials require a series of component-resolved comparators rather than only a control consisting of non-functionalized nanoparticles.

The complexity of such systems is further illustrated by chitosan-curcumin-ZnO/TiO_2_ hybrid materials developed for antibacterial applications, in which the respective contributions of the biopolymer, bioactive compound, and two metal oxides are difficult to disentangle without an appropriate series of controls [35]. Within the same broad category, lignin-encapsulated ZnO NPs have been associated with enhanced antibacterial and photocatalytic activity, suggesting that a biopolymeric matrix may preserve or amplify a material’s functional reactivity rather than merely shielding it [36]. These examples demonstrate that the presence of a biopolymer should not automatically be interpreted as a factor that passivates the nanoparticle surface or reduces toxicity.

### 4.3. Biosurfactants as Modifiers of the ZnO NP and TiO_2_ NP Interface

Basak et al. (2014) used diacetylated acidic sophorolipid both as a biostabilizer during ZnO NP synthesis and as a biofunctionalizing agent, obtaining nanoparticles with a biomolecular component associated with their surface and comparing them with non-functionalized ZnO NPs [20]. This strategy sits at the interface between synthesis and biofunctionalization: the biosurfactant participates in system formation while also shaping the interfacial properties of the final product. Consequently, the experimental design allows a biologically relevant comparison, although fully separating the surface effect from potential changes occurring during nanoparticle formation remains difficult.

A strategy more clearly separated from the synthesis step is represented by P25 TiO_2_ NPs modified with surfactin [14]. In this system, association of the biosurfactant with pre-existing nanoparticles was accompanied by measurable changes in ζ-potential and hydrodynamic size, confirming the formation of an interface distinct from that of the original P25 TiO_2_ NPs. This design is particularly valuable for causal attribution because it enables investigation of the surface effect without the interface modification being inherently coupled to TiO_2_ core formation.

Researchers have also used rhamnolipids to modify ZnO NPs, particularly to improve dispersion and interactions with microorganisms [27,37]. These studies illustrate the distinction between demonstrating that biofunctionalization modifies the nanoparticle interface and demonstrating that such modification alters its safety profile. From this perspective, biosurfactants are an important category for the present review not because they are a priori safer than synthetic modifiers but because they provide a biologically derived means of modulating the interface between a metal oxide and the exposure environment. The resulting effects must nevertheless be established experimentally for each nanoparticle-biosurfactant-biological environment combination.

### 4.4. Bioactive Molecules and Multicomponent Biofunctionalization Systems

Another biofunctionalization strategy involves associating nanoparticles with smaller biologically active molecules, such as polyphenols and other naturally occurring metabolites. Curcumin is one example encountered in the ZnO NPs literature. Unlike proteins and polysaccharides, such molecules may require a linker or a chemical activation step to achieve a sufficiently stable association. In the ZnO NPs-3-mercaptopropionic acid (MPA)–curcumin system, for example, the ZnO surface was first modified with 3-mercaptopropionic acid, after which the carboxyl groups were activated to enable covalent conjugation of curcumin [38].

This example highlights an important classification issue. The presence of a biomolecule in the final product does not imply that the entire surface architecture is biological. In systems employing synthetic linkers, the resulting interface is hybrid, and its properties may reflect contributions from both the biomolecule and the linker. Such systems are therefore useful for understanding the possibilities offered by surface engineering but require caution when attributing a biological effect exclusively to the biomolecular component of the interface.

The same issue becomes even more evident in nanoformulations combining ZnO or TiO_2_ with proteins, biopolymers, targeting molecules, and/or therapeutic agents. As the number of components increases, biofunctionalization evolves from relatively simple surface modification towards the construction of a multifunctional nanoarchitecture. For this review, these systems are informative when they allow identification of the biomolecular interface. However, their causal value is lower when the effects of the nanoparticle core, biomolecules, and additional components cannot be experimentally disentangled.

A recent strategy for TiO_2_ combines phycocyanin with polydopamine in an architecture designed for UV protection, illustrating the incorporation of a pigment biomolecule into a multicomponent surface system [39]. In the case of ZnO, biofunctionalized, defect-engineered quantum dot systems investigated for UV protection further illustrate the difficulty of attributing a safety profile specifically to the biointerface when functionalization is accompanied by changes in particle size and crystalline defect structure [24]. In such cases, the effects of biofunctionalization cannot be reliably separated from those arising from changes in the intrinsic properties of the inorganic core in the absence of appropriate comparators.

### 4.5. From “Biomolecule Presence” to a Characterized Biointerface

The diversity of the strategies described above highlights a cross-cutting methodological issue: demonstrating biofunctionalization requires more than simply confirming the presence of an organic component in the nanoparticle preparation. For proteins and peptides in particular, the amount adsorbed does not necessarily reflect preservation of biological function after surface association. Molecular orientation, surface coverage density, conformational freedom, and accessibility of active domains can determine whether biomolecules retain their function after immobilization. The literature on antimicrobial peptides emphasizes that the orientation, packing density, mobility, and conformational changes of peptides bound to nanoparticle surfaces remain insufficiently characterized, despite their potential to govern biological selectivity [25].

From this perspective, demonstration of a well-defined biointerface should, whenever possible, integrate evidence of biomolecule presence at the surface with information on the amount associated, binding stability, changes in surface charge and hydrodynamic size, and, for functional biomolecules, retention of biological activity. No single analytical method can address all these levels of characterization, and causal attribution becomes more robust when complementary methods support surface modification.

Accordingly, biofunctionalization strategies for ZnO and TiO_2_ nanoparticles span a continuum ranging from the adsorption of a single type of biomolecule onto preformed nanoparticles, which allows relatively direct assessment of surface-modification effects, to one-pot synthesis and multicomponent nanoarchitectures, in which core formation and interface construction are interdependent. This distinction is essential for data interpretation: the more biofunctionalization simultaneously alters multiple material characteristics, the more difficult it becomes to attribute a biological effect to a single surface property. Consequently, the strength of the evidence depends not only on the extent and quality of material characterization but also on the design of the comparators used to distinguish the biointerface effect from concomitant changes in the nanoparticle core.

## 5. From Physicochemical Modification to Biological and Toxicological Identity

Biofunctionalization does not simply add an organic component to ZnO or TiO_2_ nanoparticles. By modifying the solid-liquid interface, it can simultaneously alter surface charge, hydration, hydrodynamic size, aggregation state, surface accessibility and reactivity, and, depending on the nature of the nanoparticle, specific processes such as dissolution and Zn^2+^ ion release in the case of ZnO NPs or photocatalytic activity in the case of TiO_2_ NPs. From this perspective, biofunctionalized nanoparticles should not be regarded toxicologically as the same particles “plus a biomolecule” but rather as entities with an altered physicochemical identity whose properties relevant to biological exposure must be determined experimentally.

### 5.1. Surface Charge and Colloidal Stability

Changes in ζ-potential are among the most obvious consequences of biofunctionalization. The direction and magnitude of these changes depend on the initial nanoparticle surface properties, the biomolecule charge, and its degree of surface association. In some systems, the change is pronounced enough to reverse the apparent surface charge of nanoparticles.

This phenomenon is well demonstrated for TiO_2_ NPs functionalized with the cationic peptide LL-37. Increasing peptide concentration progressively increases ζ-potential, and once sufficient surface coverage is achieved, the particles acquire a net positive charge and greater colloidal stability than non-functionalized TiO_2_ NPs under the same conditions [32]. Importantly, this change is not merely an indirect indication of peptide adsorption; it also alters the electrostatic character of the interface subsequently encountered by the biological system.

A more pronounced example is biofunctionalizing P25 TiO_2_ NPs with surfactin. Unmodified P25 TiO_2_ nanoparticles exhibited a ζ-potential of +41.1 mV under the characterization conditions used. In contrast, the surfactin-containing formulations showed negative values, along with substantial changes in hydrodynamic size and isoelectric point [14]. In this case, the biosurfactant not only stabilizes the dispersion but also substantially alters the interface’s electrostatic properties. Such charge modification is toxicologically relevant because it can simultaneously affect aggregation, biomolecule adsorption, membrane association, and cellular uptake, making ζ-potential an exposure-dependent descriptor of the nano-bio interface rather than a direct predictor of toxicity.

### 5.2. Hydrodynamic Size and Aggregation

Biofunctionalization may increase the size of individual nanoparticles by adding a biomolecular component while simultaneously decreasing the apparent hydrodynamic size of aggregates in dispersion by preventing or reducing aggregation. This apparent contradiction is important for data interpretation. Core size determined by TEM or XRD and hydrodynamic diameter measured in suspension describe different properties and should not be used interchangeably.

The P25 TiO_2_ NP–surfactin system clearly illustrates this distinction. Although biofunctionalization introduces additional organic material onto the TiO_2_ NP surface, the hydrodynamic diameter of P25 TiO_2_ NPs, approximately 599 nm under the conditions investigated, decreased substantially in some surfactin-containing formulations, reaching approximately 174–289 nm depending on the component ratio and preparation method [14]. Structural and thermal analyses provided additional evidence of surfactin association with the material and interface modification.

These changes in hydrodynamic diameter, surface charge, and colloidal stability have direct consequences for biological exposure and should not be interpreted merely as formulation parameters. Aggregation modifies the number, size, and sedimentation behavior of particulate entities that reach cells or biological interfaces, thereby altering the effective particle dose relative to the nominally administered dose [18,31,40]. Conversely, improved colloidal stability can maintain a larger fraction of nanoparticles in a dispersed and biologically accessible state, potentially increasing contact with membranes, cellular uptake, or tissue penetration. Surface charge further modulates these processes by influencing both interparticle interactions and interactions with proteins, membranes, and other biological components [14,22,31,32,34]. Consequently, a reduction in aggregation or an increase in colloidal stability cannot be interpreted a priori as evidence of reduced toxicity: depending on the resulting biointerface and exposure conditions, stabilization may decrease nonspecific particle deposition or, conversely, increase biological accessibility and effective exposure.

### 5.3. Surface Accessibility and Intrinsic Reactivity

A biomolecule associated with the nanoparticle surface may act as a barrier between the inorganic core and the surrounding environment; however, this barrier is not necessarily impermeable. The density, thickness, organization, and stability of the biomolecular layer determine how much water, ions, oxygen, and other molecules can access the ZnO NP or TiO_2_ NP surface.

This aspect is particularly important for TiO_2_ NPs because photocatalytic activity depends, among other factors, on the accessibility of the surface to species present in the reaction medium. The available evidence reveals two apparently contrasting outcomes. In LL-37-coated TiO_2_ NPs, the peptide layer did not significantly suppress reactive oxygen species formation, indicating that modification of the interface was insufficient to block the photochemical processes investigated [31]. In contrast, modification of P25 TiO_2_ NPs with surfactin markedly reduced the photoreactivity of the material [14]. These two cases are particularly informative because they show that biofunctionalization does not necessarily shield or suppress inorganic-core reactivity; rather, the outcome depends on the nature and organization of the biointerface.

Crystal phase represents an additional material-specific determinant of TiO_2_ biological behavior. Comparative studies have shown that anatase and rutile may differ in their interactions with biomolecules and in the resulting cellular responses, even when major physicochemical parameters are relatively similar [41]. Mixed anatase/rutile materials may likewise exhibit biological effects that differ from those of single-phase TiO_2_, including in intestinal cell models [42]. However, the literature does not support a universal toxicity ranking of anatase, rutile, and mixed-phase TiO_2_, because phase-dependent effects interact with particle size, specific surface area, surface defects, aggregation state, surface modification, illumination conditions, and the biological model [41,43,44]. Accordingly, crystal phase should be reported and controlled as a material-specific variable when assessing how biofunctionalization modifies the biological and toxicological behavior of TiO_2_.

The same principle is supported by N-doped TiO_2_ incorporated into a chitosan hydrogel matrix. The biopolymeric matrix can modify the accessibility of biomacromolecules to the nanoparticle surface, while blue-light activation preserves sufficient photoreactivity to induce biomacromolecule degradation [45]. Thus, incorporation into a biological matrix does not necessarily imply neutralization of TiO_2_ NP reactivity.

### 5.4. ZnO NP Dissolution: A Surface- and Environment-Dependent Property

For ZnO NPs, dissolution requires particular attention because Zn^2+^ release represents an important component of their biological behavior. In principle, biofunctionalization may alter dissolution kinetics and ion release by limiting surface contact with the surrounding medium, changing surface hydration, or modifying the accessibility of protons and ligands. However, the available evidence indicates that this relationship is not straightforward and depends strongly on the environment in which the nanoparticles are evaluated.

A useful mechanistic benchmark comes from Luo et al. [32] in which ZnO NPs derived from the same starting material were modified with PEG or APTES. Although these coatings are synthetic, the experimental design allows evaluation of surface modification without intentionally changing the nanoparticle core composition. PEG reduced protein adsorption and cellular uptake; however, in artificial lysosomal fluid at pH 4.5, both coated and uncoated nanoparticles dissolved almost completely, with no significant differences between them [32]. This finding shows that a surface layer that modifies extracellular interactions does not necessarily protect the ZnO core in an acidic environment that strongly favors dissolution. Therefore, the effects of biofunctionalization on ZnO NPs must be evaluated by site and stage of exposure: stabilization in the extracellular environment and persistence within intracellular compartments are not equivalent properties.

### 5.5. Biointerface Stability: An Insufficiently Characterized Property

An important issue emerging from the literature analyzed is that many studies rigorously characterize nanoparticles immediately after biofunctionalization but provide considerably less information on the persistence of this state over time. For toxicological interpretation, however, it is not sufficient to establish that biomolecules are associated with the NP surface at the time of preparation. It is also necessary to determine whether they remain associated during storage, dispersion, exposure, and contact with biological media. Stability may be influenced by pH, ionic strength, competition with other biomolecules, dilution, and, for photoreactive TiO_2_ NPs, oxidative processes generated at the nanomaterial surface. In LL-37-TiO_2_ NP systems, for example, the surface-associated peptide largely retained its effect on particle charge even after UV exposure. However, evidence suggests partial degradation or oxidation [33]. Such information is more relevant to characterizing the biointerface under exposure conditions than characterization performed exclusively on the freshly prepared material.

Biointerface stability therefore represents an essential link between the initial physicochemical characterization and the identity of the nanoparticle that encounters the biological system. An unstable biointerface may lead to progressive exposure of the inorganic surface, desorption of biomolecules, or their replacement by components of the surrounding medium. Consequently, the toxicologically relevant identity of the nanoparticle may differ substantially from that characterized immediately after preparation.

### 5.6. The Physicochemical Identity of ZnO NPs and TiO_2_ NPs Is Environment-Dependent

Because biofunctionalization acts on materials that undergo fundamentally different transformation pathways, the physicochemical parameters determining their resulting toxicological identity are not identical for ZnO and TiO_2_ NPs. Table 1 summarizes the major mechanistic differences and their implications.

These physicochemical transformations acquire toxicological significance through their effects on biological recognition, accessibility, uptake, and effective exposure, which are considered in the following subsections. These physicochemical transformations acquire toxicological significance through their effects on biological recognition, accessibility, uptake, and effective exposure, which are considered in the following subsections.

### 5.7. From Physicochemical to Biological and Toxicological Identity

In nanotoxicology, the biological identity of a nanoparticle is dynamic and environment-dependent. Adsorption of proteins, lipids, and other biomolecules can modify the surface presented to the biological system, so subsequent interactions are no longer determined exclusively by the properties of the initial nanoparticle. For biofunctionalized nanoparticles, this process begins from an already engineered biointerface, which may subsequently be retained, supplemented, masked, or remodeled in the biological environment. Thus, the deliberately created biointerface should not be equated with the nanoparticle’s final biological identity.

In the present review, we use the term “toxicological identity” in an integrative sense to describe the toxicological profile emerging from this sequence of transformations and interactions. It is not considered an intrinsic and invariant property of ZnO or TiO_2_ NPs, nor a direct consequence of the mere presence of a biomolecule at the surface. Rather, it emerges from the interplay between physicochemical identity, the engineered and subsequently transformed biointerface, biological accessibility, and the specific exposure context. Consequently, nanoparticles with the same inorganic core may exhibit different toxicological profiles when biofunctionalization changes their interactions with biological systems.

Operationally, the toxicological identity of a nanoparticle formulation can be assessed as a multidimensional profile rather than by a single metric. Comparative assessment should integrate (a) effective exposure, including dispersed, sedimented, dissolved, and internalized fractions where relevant; (b) transformation of the nanoparticle and its biointerface, including aggregation, dissolution, ligand retention or exchange, and corona formation; (c) biological accessibility, assessed through cellular uptake, translocation, and biodistribution; and (d) hazard responses, including cytotoxicity, oxidative stress, inflammation, genotoxicity, and other mechanism-relevant molecular endpoints. Where targeting is intended, responses in target and non-target systems should additionally be compared. Toxicological identities of different formulations can therefore be compared quantitatively using matched nanoparticle cores, equivalent exposure conditions, and the same endpoint panel, expressing changes relative to the corresponding non-biofunctionalized formulation.

Biofunctionalization may therefore modify toxicological outcomes without eliminating the intrinsic reactivity of the inorganic core. It can reduce nonspecific contact or internalization, increase biological accessibility, or redirect nanoparticles toward selected targets. Evaluation should consequently trace the sequence surface modification–biological interaction–effective exposure at the target site–toxicological response, rather than relying solely on material characterization before and after functionalization.

### 5.8. Interactions with Biological Membranes

The cell membrane is one of the first biological structures directly affected by changes in nanoparticle surface properties. Biofunctionalization can therefore modify both the extent of membrane interaction and the preference for specific membrane compositions.

Studies using peptide-coated TiO_2_ NPs provide some of the clearest evidence of this effect. Non-functionalized TiO_2_ NPs exhibit relatively weak interactions with model anionic membranes. In contrast, coating with the cationic peptide LL-37 markedly enhances their association with membranes rich in anionic phospholipids, characteristic of bacterial membrane models [25]. This effect is accompanied by more pronounced membrane disruption, while interactions with membranes mimicking mammalian cells remain considerably weaker. Thus, surface modification alters nanoparticles’ preference for different types of biological membranes.

This selectivity can also be tuned through the structure of the biomolecules used for biofunctionalization. In the case of the peptides KYE21 and WWWKYE21, the introduction of additional tryptophan residues modifies their affinity for the TiO_2_ surface and promotes interactions with bacterial-like membranes [33]. In this context, biomolecules function as recognition elements that transform the oxide surface from a relatively nonspecific interface into one with distinct biological preferences.

A similar phenomenon, although mediated by a different class of biomolecules, is suggested by sophorolipid-biofunctionalized ZnO NPs. Compared with non-functionalized ZnO NPs, the modified nanoparticles induced more pronounced disruption of microbial membranes and greater release of intracellular components [20]. In this case, biofunctionalization does not attenuate biological activity; rather, it enhances nanoparticle interactions with the microbial target.

### 5.9. Cellular Uptake and Biological Accessibility

Changes in the biointerface can substantially alter cellular accessibility and uptake. The study by Vian et al. [18] demonstrates that biofunctionalization that appears favorable from a colloidal perspective may increase cellular accessibility. In HT-29 intestinal cells, the proportion of TiO_2_ NPs detected intracellularly was approximately 28% for uncoated particles, compared with approximately 48% for gelatin-coated TiO_2_ NPs and 70% for β-lactoglobulin-coated particles [18]. Thus, protein coating may prevent aggregation and stabilize nanoparticles, increasing the fraction of material available to interact with cells.

Thus, the biocompatibility of a coating biomolecule does not necessarily imply reduced cellular exposure, because improved dispersion may increase the effective particulate dose available to cells.

Studies of synthetic surface modification of ZnO NPs illustrate the opposite. Although PEG is not a biomolecule and the study by Luo et al. [32] is considered here only as a mechanistic benchmark, PEGylation reduced protein adsorption and cellular uptake, accompanied by reduced ZnO cytotoxicity. This experiment highlights a relationship directly relevant to biofunctionalization: surface properties → biomolecular interactions → cellular uptake → intracellular exposure. Modifying the first element in this sequence can alter toxicity even when the nanoparticle core’s intrinsic reactivity remains unchanged.

### 5.10. Targeting Biological Structures

Biofunctionalization can extend beyond nonspecific modulation of cellular uptake, enabling nanoparticles to preferentially recognize specific biological structures. This clearly demonstrates that toxicological identity is not determined exclusively by a material’s intrinsic toxicity.

In the TiO_2_-dihydrolipoamide dehydrogenase (DLDH)-Arg-Gly-Asp (RGD) system, the RGD motif was introduced to enable recognition of integrins, such as αvβ3, which are highly expressed in B16F10 melanoma cells. Without UV activation, the system showed no significant cytotoxicity. Following UVA irradiation, however, TiO_2_ NPs and TiO_2_-DLDH reduced tumor and control cell populations to a comparable extent, whereas TiO_2_-DLDH-RGD caused a reduction of more than 95% in B16F10 cells without a comparable increase in the effect on HEK293 cells [19]. The biomolecular coating did not “detoxify” the TiO_2_ NPs; rather, it redirected their phototoxic effect towards the cell population expressing the molecular target.

A similar rationale applies to the TiO_2_-BSA-PyBA system, where surface modification increased cytotoxicity compared with unmodified nanoparticles. Still, the effect was more pronounced in tumor cells than in normal cells [15]. From a safety perspective, such an outcome cannot be adequately described using a single category of “more toxic” or “less toxic.” Instead, the relevant parameter becomes the selectivity of the toxicological effect. Direct interactions between metal oxide surfaces and biomolecules may also constitute a component of toxicological identity, independently of classical mechanisms based exclusively on ROS. Using polyoxometalates as molecular models of metal oxides, Svensson et al. [46] demonstrated that charge-transfer interactions and hydrogen bonding with nucleosides can facilitate N-glycosidic bond cleavage and their subsequent deglycosylation. However, a TiO_2_ model based on TiBALDH did not produce the same reaction under the experimental conditions used, highlighting that such mechanisms cannot be generalized across different metal oxide surfaces [46]. These examples show that selectivity is itself a dimension of toxicological identity, because changes in nanoparticle recognition, uptake, or localization can redirect effective exposure toward specific biological targets without necessarily altering the intrinsic reactivity of the inorganic core.

However, selectivity should not be interpreted as a substitute for conventional safety assessment. No universal quantitative selectivity window can presently be defined for biofunctionalized ZnO or TiO_2_ nanoparticles. An acceptable margin must be established for the intended application by comparing target and non-target responses across relevant exposure levels, while ensuring that non-target effects remain within applicable toxicological and regulatory limits. Thus, selectivity is best considered an additional comparative dimension of safety rather than an independent regulatory threshold.

**Figure 2:**
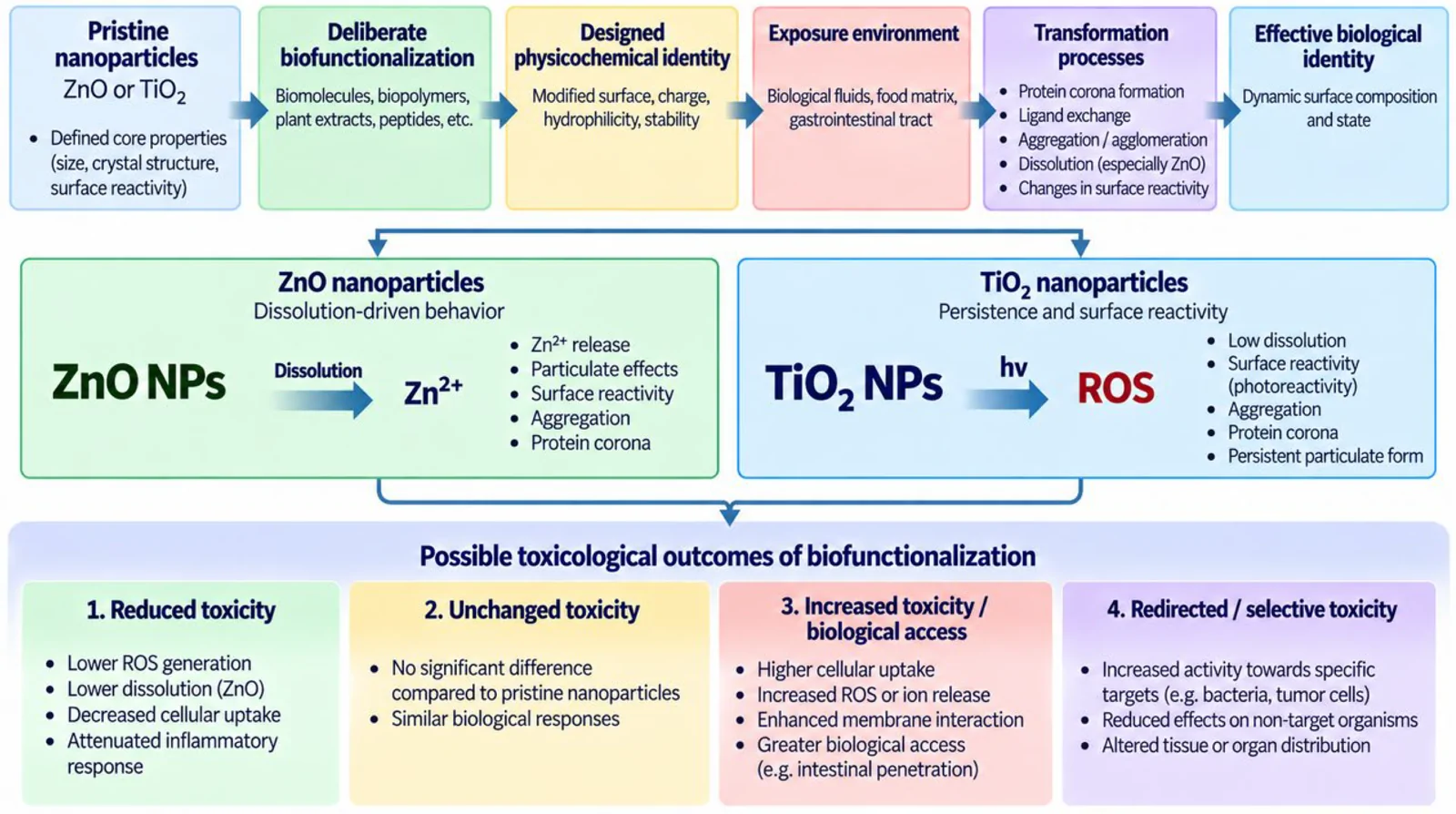
Biofunctionalization-driven changes in the biological and toxicological identity ZnO and TiO_2_ nanoparticles.

Figure 2 integrates these interdependent processes into a single trajectory from deliberate biofunctionalization to the evolving biological and toxicological identity of the nanoparticle.

This trajectory remains material-specific: ZnO requires consideration of dissolution and Zn^2+^ release alongside particle-specific effects, whereas the greater persistence of TiO_2_ shifts the emphasis toward aggregation, corona formation, surface reactivity, and photoreactivity.

Overall, biofunctionalization outcomes are not limited to reduced toxicity. Depending on how the engineered biointerface modifies exposure and nano–bio interactions, toxicity may decrease, remain largely unchanged, increase because of enhanced biological accessibility, or be redirected toward specific biological targets. The central consequence is therefore a reprogramming of nano–bio interactions rather than simple detoxification. We refer to this dynamic, context-dependent outcome as the evolving toxicological identity of biofunctionalized ZnO and TiO_2_ nanoparticles.

## 6. Does Biofunctionalization Make ZnO and TiO_2_ Nanoparticles Safer?

The comparative evidence analyzed does not support a simple yes-or-no answer. Although biofunctionalization is often associated with terms such as biocompatibility, reduced toxicity, or safe-by-design, studies that can distinguish the effects of biomolecular surface modification from those arising from other nanoparticle characteristics remain relatively limited. Moreover, their findings do not indicate a consistent direction of effect. Biofunctionalization may reduce toxicity, produce minor or coating-dependent changes, increase biological accessibility or activity toward specific targets, and, in many cases, alter the selectivity of the effect without diminishing the nanoparticle’s intrinsic reactivity. Therefore, being “safer” is not a general property of biofunctionalized nanoparticles but rather an outcome that depends on the nature of the biointerface, the material, the biological target, and the exposure context. Given the limited number of studies meeting the CORE criteria, the conclusions drawn from the comparative evidence should be interpreted according to their level of direct experimental support. The most consistently supported conclusion is that biofunctionalization does not produce a uniform reduction in toxicity: depending on the engineered biointerface and exposure context, it may reduce non-target effects, increase biological accessibility, preserve intrinsic reactivity, or redirect effects toward selected targets. By contrast, conclusions concerning specific biofunctionalization strategies, quantitative safety advantages, and their persistence across exposure environments remain preliminary and formulation-dependent. The strength of the comparative evidence is also unequal between the two oxides, with more direct evidence currently available for TiO_2_ than for ZnO. Consequently, TiO_2_-derived findings concerning peptide-mediated targeting, preservation or redirection of photocatalytic activity, and modulation of interactions with biological membranes should not be extrapolated to ZnO. Conversely, conclusions concerning ZnO dissolution and Zn^2+^ release are material-specific and cannot be transferred to TiO_2_. Cross-material interpretation is therefore limited to broader principles of biointerface remodeling, exposure-dependent transformation, effective biological exposure, and the need for appropriate comparators. Table 2 summarizes comparative studies that provide the strongest evidence for a direct effect of biofunctionalization on the toxicological behavior of ZnO and TiO_2_ nanoparticles.

### 6.1. Evidence for Reduced Toxicity

The strongest evidence for reduced toxicity comes from studies that evaluated functionalized and non-functionalized nanoparticles under comparable conditions. For ZnO NPs, Karthikeyan et al. [17] showed that the outcome depends on the biopolymer used. In HepG2 cells, the IC_50_ of non-functionalized ZnO NPs was approximately 64 µg/mL, whereas chitosan functionalization increased the IC_50_ to approximately 128 µg/mL, indicating reduced cytotoxicity. In contrast, alginate-associated ZnO NPs exhibited an IC_50_ value close to that of non-functionalized ZnO NPs [17]. This experiment is particularly relevant because it shows that reduced toxicity cannot be attributed generically to the presence of a biopolymer: two different biofunctionalization strategies applied to the same nanoparticle type can produce different toxicological outcomes.

Reduced toxicity towards host cells has also been observed for ZnO NPs encapsulated with chitosan derivatives. Compared with non-functionalized ZnO NPs, the nanocomposites exhibited lower cytotoxicity while retaining a substantial proportion of their antiviral activity [16]. In this case, the advantage of biofunctionalization lies not only in reducing toxicity but also in improving the balance between antiviral activity and adverse effects on host cells.

For TiO_2_ NPs, the surfactin-based system provides a relevant example, while also cautioning against generalization. Biofunctionalization of P25 TiO_2_ markedly reduced photoreactivity and decreased toxicity in some of the models tested. For example, the EC_50_ in the algal model increased from approximately 1.4 mg/L for P25 TiO_2_ to approximately 18 mg/L for the biofunctionalized material [14]. However, the protective effect was not uniform across biological models. In RAW264.7 macrophages, at high concentrations, the surfactin-containing formulation induced a more pronounced adverse response, while free surfactin itself exhibited concentration-dependent effects. These findings show that reduced toxicity in one biological model cannot automatically be extrapolated to other cell types or organisms.

### 6.2. Absence of a Toxicity-Reducing Effect

A neutral outcome is as informative as a protective one. It demonstrates that the mere presence of a biomolecule on the NP surface is not sufficient to modify the toxicological effect. The comparison between chitosan- and alginate-functionalized ZnO NPs illustrates this point. While chitosan reduced cytotoxicity in the HepG2 model, alginate did not produce a comparable change relative to non-functionalized ZnO NPs [17]. Therefore, the observed effect cannot be explained by a simple assumption that any biopolymeric layer shields the ZnO surface and consequently reduces toxicity.

Studies of synthetic surface modification support the same conclusion mechanistically. Luo et al. [30] observed that PEGylation of ZnO NPs reduced toxicity, whereas APTES modification resulted in cytotoxicity like that of uncoated ZnO NPs. Although this study does not represent biomolecular biofunctionalization and is considered here only as a surface-engineering benchmark, it confirms that the nature of the surface modifier, rather than the mere presence of a coating, is a key determinant of the biological effect.

### 6.3. Increased Biological Accessibility and Effect

Biofunctionalization can also produce an outcome opposite to that intended by a conventional detoxification strategy: increased biological accessibility of the nanoparticle.

One of the clearest examples is provided by TiO_2_ NPs coated with food proteins. Compared with uncoated particles, association with gelatin and, particularly, β-lactoglobulin improved dispersion and increased the uptake of TiO_2_ NPs by HT-29 intestinal cells [18]. This finding does not, by itself, demonstrate increased toxicity. Still, it highlights an essential consideration for safety assessment: a nanoparticle that is better dispersed and coated with a biocompatible molecule may become more biologically accessible.

An increased biological effect is even more evident for sophorolipid-biofunctionalized ZnO NPs. Compared with non-functionalized ZnO NPs, the biofunctionalized material produced more pronounced inhibition of *Salmonella enterica* and *Candida albicans* and greater disruption of membrane integrity [20]. In an antimicrobial context, this is desirable; from a toxicological perspective, however, it shows that biofunctionalization can potentiate, rather than merely attenuate, a nanoparticle’s ability to exert biological effects on a susceptible target.

### 6.4. Redirection of Toxicity and Increased Selectivity

Perhaps the most interesting finding in the comparative analysis is that several CORE studies cannot be adequately described within a simple “more toxic-less toxic” framework. In these cases, biofunctionalization shifts the distribution of biological effects across targets.

This is particularly evident in studies on TiO_2_ NPs functionalized with antimicrobial peptides. Association of LL-37 with TiO_2_ NPs largely preserves the photoreactive capacity of the nanoparticles while increasing their affinity for anionic structures and bacterial membranes [25]. Modifying the peptide by introducing additional tryptophan residues further tunes interactions with bacterial-like membranes [33]. In another system, LL-37 associated with TiO_2_ NPs enhances interactions with lipopolysaccharide (LPS) and lipoteichoic acid (LTA) and helps reduce monocyte activation induced by these microbial components [34]. Thus, biofunctionalization does not render TiO_2_ NPs inert; rather, it reconfigures their interactions with different biological targets.

The evidence is even more explicit in the TiO_2_-DLDH-RGD system. Following UVA activation, non-functionalized nanoparticles and those associated with DLDH alone affected both melanoma and control cells. Introducing the RGD motif, however, reduced the B16F10 cell population by more than 95%, which express high levels of αvβ3 integrin, without a corresponding increase in the effect on HEK293 cells [19]. The phototoxic effect was therefore preferentially redirected towards the target cell population rather than eliminated.

The TiO_2_-BSA-PyBA system illustrates the same principle. At 200 µg/mL, the reported cytotoxicity towards DLA tumor cells increased from approximately 14.9% for unmodified TiO_2_ NPs to approximately 36.9% for the functionalized material. In contrast, the effect on normal splenic cells increased from approximately 6.0% to 13.7% [15]. Quantitatively, the biofunctionalized material was more cytotoxic in both models, but the difference in response between tumor and normal cells was substantial. Classifying the material simply as “more toxic” would therefore overlook the change in biological selectivity produced by functionalization.

Toxicological selectivity should therefore be considered a distinct dimension of biofunctionalization effects. In applications where nanoparticles are intended to retain antimicrobial, antiviral, or therapeutic activity, the realistic objective is not zero toxicity but rather maximizing the effect on the intended target while minimizing adverse effects on non-target cells or organisms.

### 6.5. The Problem of Biogenic Nanoparticles Reported as Safe

Much of the literature attributes favorable properties to nanoparticles produced through biological or “green” synthesis. These studies are relevant to the present review but require a different interpretation from that applied to direct comparative experiments.

ZnO NPs synthesized using plant extracts, fungi, or cyanobacteria may exhibit substantial biological activity and, in some models, relatively low toxicity towards normal cells. For example, ZnO NPs produced using *Arthrospira platensis* showed a considerable difference in cytotoxicity between Caco-2 cells and normal WI38 cells [22]. Other biogenic systems have been reported to exhibit low acute oral toxicity or favorable biological effects [28,30].

However, other findings challenge the implicit association between biological origin and safety. ZnO produced through a biological approach induced measurable toxicity in *Cyprinus carpio*, demonstrating that the biological origin of the material or the presence of biologically derived components associated with its surface does not guarantee the absence of ecotoxicological effects [49,53]. Other recent formulations, such as ZnO NPs produced using *Frankenia hirsuta*, have been associated with favorable anti-inflammatory effects, but without a comparator that would allow the contribution of the surface-associated biomolecular component to be distinguished from the properties of the material as a whole [54].

However, without a control of comparable ZnO NPs lacking the biologically derived surface component, these findings do not show that surface-associated biomolecules drive the observed toxicological profile. Biogenic synthesis can simultaneously alter particle size, morphology, size distribution, crystalline defects, aggregation behavior, and surface chemistry. The observed biological profile therefore belongs to the biogenic material as a whole and cannot automatically be attributed to surface biofunctionalization.

### 6.6. What Do Comparative Studies Demonstrate Overall?

Taken together, the comparative studies indicate that the characteristic outcome of biofunctionalization cannot be reduced to detoxification but is better described as a reconfiguration of nanoparticle-biological system interactions. The toxicological outcome depends on how the newly established biointerface modifies exposure, recognition, and access to the biological target. Biofunctionalization may reduce toxicity in one system, have a limited or neutral effect in another, increase biological accessibility, or redirect the effect towards specific targets.

The answer to the question posed in the title of this review is therefore conditional: biofunctionalization can make ZnO and TiO_2_ nanoparticles safer, but safety is not an intrinsic property of the biofunctionalization process. Rather, researchers must demonstrate it experimentally for each nanoparticle-biointerface-exposure environment-biological target combination. In this context, the relevant criterion is not simply the presence of a biomolecule at the nanoparticle surface but how much the biointerface favorably shifts the balance between exposure, effects on the intended target, and effects on non-target systems.

## 7. Biofunctionalization in Complex Biological Environments: How Stable Is Engineered Identity?

### 7.1. The Biomolecular Corona as a Secondary Surface Modifier

Biomolecular corona formation is one of the most important transformations nanoparticles undergo in biological environments. The adsorption of proteins and other biomolecules modifies the nanoparticle-environment interface and can influence aggregation, cellular recognition, uptake, and nanoparticle distribution. For a biofunctionalized nanoparticle, however, the corona forms on a surface that already possesses an engineered physicochemical and biological identity.

From this perspective, biofunctionalization and corona formation are not independent phenomena. The former may determine which biomolecules are subsequently adsorbed, in what amounts, and with what affinities. In contrast, the acquired corona may, in turn, modify the functional accessibility of the deliberately introduced biomolecules. The final surface therefore emerges from the interaction between the engineered biointerface and the biological environment. Consequently, characterization of a nanoparticle immediately after biofunctionalization is insufficient to define the interface that will be presented to the biological system.

Studies of TiO_2_ NPs exposed to extracellular proteins illustrate the importance of this transformation. Alijagic et al. [55] showed that protein corona formation on TiO_2_ NPs modifies nanoparticle interactions with phagocytes, including their aggregation and internalization. In another system, biomolecule adsorption reduced nanoparticle association with fungal spores and protected against nanoparticle toxicity, including that of ZnO NPs [56]. This finding is particularly relevant because it reverses some of the deliberately engineered biofunctionalization effects discussed above. Whereas an engineered surface may increase affinity for a particular target, an acquired corona may shield the nanoparticle surface and reduce contact with that target.

A relevant example of the transition from an initially modified surface to one remodeled by the biological environment is provided by metal oxide nanoparticles synthesized using cannabidiol. Exposure to cell culture medium resulted in protein corona formation and changes in hydrodynamic size, illustrating the transition from an engineered interface to an acquired one [40]. At the ecotoxicological level, researchers have comparatively investigated the relationship between corona composition and biological response in engineered nanoparticles in *Paracentrotus lividus*, further supporting the relevance of acquired identity in determining biological responses [48].

### 7.2. Final Identity Depends on Surface History

A less intuitive aspect is that the acquired biological identity depends not only on the composition of the final environment but also on the order in which the nanoparticle encounters its different components. This dependence on exposure history has direct implications for biofunctionalization.

Shakiba et al. [50] showed that simultaneous and sequential exposure of TiO_2_ NPs to components of organic matter and BSA do not produce equivalent surfaces. Sequential exposure may lead to layered adsorbed structures. In contrast, simultaneous exposure favors different interfacial configurations, determined in part by interactions among biomolecules before their adsorption onto the nanoparticle surface [50]. Thus, even if nanoparticles ultimately reach an environment with the same overall composition, the pathway they take may produce a different biointerface.

Kim and Doudrick [49] support this concept in their study of TiO_2_ NPs, BSA, and oxalate. The prior presence of a molecule adsorbed onto the nanoparticle surface subsequently altered BSA adsorption, protein conformation, and particle aggregation behavior [49]. These findings suggest that the surface retains a form of “interfacial memory,” whereby previous interactions condition subsequent ones. The dynamic nature of the nano–bio interface also has important methodological implications. Protein-corona formation involves time-dependent adsorption, desorption, exchange, and reorganization of biomolecules, so the composition and organization of the acquired interface may evolve throughout exposure [57,58]. Importantly, procedures used to isolate nanoparticle–corona complexes can themselves affect particle aggregation or corona composition, potentially altering the interface being characterized [59]. In situ or minimally perturbing approaches are therefore particularly valuable for resolving dynamic changes in the nano–bio interface under conditions closer to the actual exposure environment [57,59].

Based on these considerations and the exposure-dependent transformations discussed above [21,40,49,50], we propose that biointerface stability should be evaluated as a time- and environment-dependent property rather than as a single characteristic of the initial formulation. At minimum, the designed biointerface should be characterized immediately after functionalization, after dispersion in the relevant exposure medium, during early biological exposure, and at a later exposure stage. Where relevant, additional measurements should follow major transformation steps, such as simulated gastrointestinal digestion or exposure to acidic intracellular conditions. Complementary measurements of hydrodynamic diameter, ζ-potential, aggregation state, ligand retention or exchange, and corona composition should be combined with material-specific endpoints such as Zn^2+^ release for ZnO or surface reactivity and photoreactivity for TiO_2_. Particular attention should be given to the retention and exchange of functional ligands and to major proteins, lipids, or other biomolecules that may determine biological recognition. Whenever experimentally feasible, ligand-exchange and corona-remodeling kinetics should be followed rather than assessed only at a single endpoint, and in situ or minimally perturbing approaches should complement ex situ analyses [57,59].

### 7.3. The Biological Matrix Can Modify Both Nanoparticles and Biomolecules

Interactions between nanoparticles and biological environments are bidirectional. Biomolecules not only modify the nanoparticle surface, but the nanoparticle itself can also alter the structure of adsorbed biomolecules. This is particularly relevant to biofunctionalization because a protein layer’s function depends not only on its presence but also on preserving its conformation and functional accessibility.

Studies involving albumin illustrate the variability of these effects. Interaction of ZnO nanorods with serum albumin can induce conformational changes in the protein, and a concentration-dependent decrease in the α-helical content of human serum albumin has been reported for ZnO nanostructures [60]. Other systems, however, indicate more limited structural changes. For TiO_2_ NPs, interaction with BSA may locally affect protein structure without necessarily causing global denaturation or precipitation [61]. Similarly, in a ZnO-BSA system, local conformational changes consistent with the formation of a relatively stable protein interface have been described without complete loss of secondary structure [47].

Spectroscopic data on interactions between BSA and ZnO/Ag nanoparticle systems provide further evidence that protein adsorption involves reciprocal changes in both the interface and the biomolecule. However, such mixed systems do not allow the observed effects to be attributed exclusively to the ZnO component [62]. Therefore, demonstrating a protein’s persistence at the nanoparticle surface does not equate to preserving its biological function.

### 7.4. The Gastrointestinal Tract as an Environment of Sequential Transformation

The stability of the biointerface becomes particularly important in the context of oral exposure to biofunctionalized nanoparticles. Ingested nanoparticles do not encounter a single biological environment but pass sequentially through the food matrix, gastric environment, and intestinal compartments, which differ markedly in pH, ionic strength, enzymatic composition, and bile salt concentration. Each stage may modify nanoparticle aggregation and surface composition before the particles reach the intestinal epithelium.

For TiO_2_ NPs used in food-related contexts, Coreas et al. [21] showed that the biological corona evolves during simulated digestion and strongly depends on food composition. A lipid-rich matrix produced a more pronounced corona and altered the aggregation state compared with other food conditions. These findings show that the ingestion matrix itself helps define the nanoparticle’s biological identity. Consequently, the same nanoparticle ingested in water or in a lipid-rich food may present a different biointerface and aggregation state by the time it reaches the intestinal epithelium.

### 7.5. Aggregation and Dissolution Modify the Biologically Effective Nanoparticle Dose

Transformations in complex environments influence not only the nanoparticle surface but also the amount and form of material that becomes biologically available. From this perspective, the nominal nanoparticle concentration does not equal the dose that reaches the cellular or tissue surface. For ZnO NPs, this distinction is further complicated by dissolution, because exposure may involve both a particulate fraction and Zn^2+^ ions released into the exposure medium.

To quantify specifically how biofunctionalization modifies ZnO dissolution kinetics, future studies should use paired formulations prepared from the same ZnO core, with and without biofunctionalization, and expose them in parallel under identical conditions. Dissolved Zn should be quantified at multiple time points after appropriate separation of the dissolved and particulate fractions, allowing dissolution profiles and kinetic parameters to be compared directly between formulations [63,64]. Measurements should be performed in media representative of the intended exposure and, where relevant, across physiologically relevant pH conditions, because ZnO dissolution is strongly environment-dependent [11]. The physicochemical state of the particles, including hydrodynamic size, aggregation, ζ-potential, and retention of the functionalizing layer, should be monitored in parallel to determine whether changes in dissolution coincide with remodeling of the biointerface. An equivalent soluble Zn control should additionally be included in biological experiments to distinguish effects associated with released Zn^2+^ from those attributable to the remaining particulate fraction. Such a paired, time-resolved design would allow the effect of biofunctionalization on ZnO dissolution to be expressed quantitatively through, for example, differences in the fraction of Zn dissolved over time and, where supported by the data, dissolution rate constants.

Vaccari et al. [51] demonstrated this complexity for ZnO NPs and TiO_2_ NPs subjected to simulated gastrointestinal digestion followed by fermentation with human intestinal microbiota. Effects on the microbiota and metabolome did not always follow a monotonic relationship with nominal concentration; for ZnO NPs, under certain conditions, the lower concentration produced more pronounced changes than the higher concentration, a phenomenon the authors partly attributed to aggregation [51]. This type of response demonstrates that toxicological assessment based exclusively on the administered mass may be misleading when the dispersion state and, in the case of ZnO NPs, the relative contributions of the particulate and dissolved fractions change during exposure.

Molecular-level responses provide an additional dimension for understanding the toxicological effects of ZnO and TiO_2_ nanoparticles. For foodborne TiO_2_, Yan et al. (2022) [52] reported alterations in intestinal barrier-related molecular pathways, while Lei et al. (2026) [65] linked prolonged exposure to disruption of microbiota–host signaling and altered regulation of mucin-related processes. Molecular responses have also been reported for ZnO NPs, including changes in pathways associated with oxidative stress, mitochondrial dysfunction, autophagy, and apoptosis [66]. These findings demonstrate that the biological effects of ZnO and TiO_2_ extend beyond cellular-level endpoints to molecular regulatory pathways. However, such evidence remains scarce in studies directly comparing the same nanoparticle before and after deliberate biofunctionalization. Future comparative studies should therefore integrate targeted gene-expression analyses and, where appropriate, transcriptomic, proteomic, and metabolomic approaches to determine whether biofunctionalization changes not only the magnitude of toxicity but also the molecular pathways through which biological effects develop.

Taken together, this evidence indicates that biofunctionalization should be evaluated as an initial state of the nanoparticle interface rather than as a static and permanent property of the nanoparticle. The engineered biointerface may persist, become masked, undergo remodeling, or be partially replaced during exposure, and these transformations may alter aggregation, dissolution, biological recognition, and the effective dose reaching the target. Safety assessment of biofunctionalized nanoparticles should therefore characterize not only the material before exposure but also, whenever possible, the identity it acquires within the relevant biological environment.

## 8. Implications for Oral Exposure and Food Safety

In this review, oral exposure matters not only for documenting the toxicity of ZnO and TiO_2_ after ingestion but also for identifying the processes and biological targets biofunctionalization would need to modify to provide a genuine safety advantage. Direct comparative evidence for biofunctionalized nanoparticles following oral exposure remains limited. Therefore, the available studies on non-functionalized ZnO and TiO_2_ nanoparticles are used here to define the major barriers that a biofunctionalization strategy must overcome: gastrointestinal transformation of the biointerface, interactions with the microbiota and intestinal barrier, systemic bioavailability, and, for ZnO NPs, the variable contribution of Zn^2+^. Building on the biointerface transformations described in Section 8, the relevant question becomes whether the engineered toxicological advantage persists at the major targets of oral exposure: the microbiota and intestinal barrier, systemic compartments, and, in a material-specific manner, the pathways associated with ZnO dissolution or TiO_2_ persistence.

Addressing this gap requires a tiered experimental design comparing the same nanoparticle core in pristine and biofunctionalized forms throughout oral exposure, from food-matrix interaction and simulated gastrointestinal digestion to intestinal in vitro models, ex vivo systems, and targeted in vivo validation. Relevant endpoints should include biointerface transformation, barrier integrity, cellular uptake and translocation, microbiota responses, absorption, and biodistribution, together with ZnO dissolution or TiO_2_ persistence. Co-exposure should also be considered, since orally encountered nanoparticles may interact with food components, additives, contaminants, or pharmaceuticals. Experimental studies show that ZnO interactions with albumin can modify intestinal transport and toxicokinetics [67], while ZnO–dimethoate co-exposure altered biodistribution and toxicity [68]. For TiO_2_, co-exposure with fipronil affected intestinal metabolomic and microbiota responses [69,70], whereas combined exposure with acrylamide influenced oxidative stress, apoptosis, and genotoxicity [71]. Future studies should therefore determine whether the toxicological advantage conferred by biofunctionalization persists across gastrointestinal transformation and under realistic multicomponent exposure conditions.

### 8.1. The Intestinal Barrier and Microbiota as an Integrated System of Toxicological Interactions

The intestinal epithelium represents the first major cellular barrier following ingestion; however, nanoparticle effects cannot be reduced to direct cytotoxicity towards enterocytes. Exposure may alter intestinal permeability, intercellular junction integrity, the mucus layer, and inflammatory responses, and these effects may develop concurrently with changes in the microbiota and microbial metabolites. The intestinal barrier and microbiota should therefore be considered interdependent components of the same exposure and toxicological response system.

For biofunctionalized nanoparticles, this integrated system represents a particularly stringent test of the engineered toxicological advantage. Surface modification may alter dispersion, biomolecule adsorption, interactions with mucus, access to the epithelium, and interactions with commensal microorganisms. Consequently, a reduction in cytotoxicity observed in a simple cell culture model cannot be directly extrapolated to oral safety.

For TiO_2_ NPs, in vivo studies support this integrated perspective. In juvenile mice, oral exposure to micro- and nanoscale TiO_2_ induced alterations in the gut microbiota, intestinal inflammation, increased intestinal permeability, and disruption of tight junction proteins, with more pronounced effects at the same dose for the nanoscale form [52]. More recent evidence extends the mechanism beyond direct nanoparticle-epithelium interactions. Prolonged exposure to food-grade TiO_2_ NPs was associated with reductions in certain beneficial bacteria, alterations in microbial metabolites, and disruption of signaling pathways involved in maintaining the mucus layer. Microbiota transfer and supplementation with indole-3-lactic acid supported the microbiota’s contribution to barrier impairment [65]. Thus, the microbiota is not merely an additional exposure target but may actively participate in the mechanism by which nanoparticles affect intestinal integrity.

This dimension is particularly relevant to nanoparticles with antimicrobial activity, especially ZnO NPs. However, nominal concentration alone cannot predict effects on the microbiota. Following simulated gastrointestinal digestion and fermentation with human faecal microbiota, ZnO NPs and TiO_2_ NPs produced distinct changes in microbial community structure and the metabolome, and dose-response relationships were not always linear [51]. For ZnO NPs, the more pronounced effects observed under certain conditions at lower rather than higher concentrations were associated with, among other factors, differences in aggregation and, consequently, material availability.

The implication for biofunctionalization is therefore twofold. Surface modification may reduce nonspecific interactions and direct toxicity, but by improving dispersion or stability, it may simultaneously increase the biologically accessible fraction available to the intestinal epithelium and microbiota. In nanoformulations designed for antimicrobial activity, the technologically intended property may itself become a source of undesirable effects on the commensal microbiota. Demonstrating an oral toxicological advantage therefore requires evaluating the nanoparticle-microbiota-microbial metabolite-intestinal barrier axis after the biointerface transforms under gastrointestinal conditions, rather than merely comparing the cytotoxicity of the initial formulations.

### 8.2. Persistence of the Biofunctionalization Advantage Beyond the Intestinal Barrier

If biofunctionalization is proposed as a strategy to reduce toxicity, its advantage must be demonstrated beyond the intestinal barrier. Changes in dispersion, dissolution, absorption, or interactions with biomolecules may influence the amount and form of the material that crosses the intestinal compartment and, consequently, its effects on distant organs. The available data for non-functionalized nanoparticles therefore define the systemic risks against which future biofunctionalized formulations should be compared.

In a canine model, oral administration of ZnO NPs was associated with hepatic Zn accumulation, oxidative stress, mitochondrial dysfunction, and alterations in autophagic and apoptotic processes. These effects were accompanied by substantial changes in the metabolome and gut microbiota, supporting the involvement of a gut-liver axis in the response to exposure [66]. In such a scenario, a protective biointerface should reduce not only local effects but also systemic exposure to biologically active forms of the material or systemic metabolic disturbances, without increasing particle translocation through excessive stabilization.

In another experimental mouse model, repeated intragastric exposure to ZnO NPs was associated with behavioral alterations, together with changes in the gut microbiota and metabolic profiles in serum and the hippocampus [72]. These associations do not, by themselves, establish a causal microbiota-brain relationship, but they demonstrate that toxicological assessment of oral exposure should not be restricted to the initial site of contact.

Effects beyond the intestines are not limited to ZnO NPs. Although arising from an ecotoxicological context distinct from oral exposure in mammals, observations in fish support the broader relevance of gut-liver communication in the response to TiO_2_ NPs [73].

### 8.3. ZnO: Can Biofunctionalization Simultaneously Control Particulate Exposure and Zn^2+^ Release?

ZnO NPs require an approach distinct from that applied to TiO_2_ NPs because of their capacity to dissolve. In the gastrointestinal environment and subsequently within biological compartments, the relative proportions of particulate ZnO and Zn^2+^ may vary considerably, complicating the attribution of observed effects to a single exposure form. Direct comparisons between ZnO NPs and equimolar ZnSO_4_ show that dissolution contributes substantially to the biological response, although it does not fully account for nanoparticle effects [11].

This duality has direct consequences for biofunctionalization. A biointerface may reduce direct contact between the ZnO surface and cells or modify particle aggregation and accessibility without necessarily preventing subsequent dissolution in acidic compartments. Conversely, particle stabilization could reduce immediate Zn^2+^ release while prolonging the persistence and biological accessibility of the particulate fraction. Demonstrating reduced toxicity of biofunctionalized ZnO therefore requires assessing the particulate fraction, released Zn^2+^, and the biologically effective dose simultaneously (Table 1). Reducing a single mechanism does not automatically reduce overall risk.

### 8.4. TiO_2_: Biofunctionalization of a Persistent Particle Requires Evaluation of the Interface and Long-Term Exposure

For TiO_2_ NPs, for which dissolution does not represent a mechanism comparable to that of ZnO NPs, biofunctionalization acts on a relatively persistent particle. The toxicological advantage therefore depends largely on the persistence of the modified interface and on how it controls aggregation, corona formation, interactions with mucus and microbiota, access to the epithelium, and potential absorption. This distinction from ZnO NPs, summarized in Table 1, means that the safety of biofunctionalized TiO_2_ NPs cannot be inferred solely from reduced acute toxicity of the initial formulation.

Repeated oral exposure to TiO_2_ NPs has been associated with systemic metabolic alterations and oxidative stress markers. In a 90-day study in rats, administration of nanoscale TiO_2_ produced, at higher doses, changes in body weight and triglyceride levels, extensive alterations in the metabolomic profile, and changes in oxidative stress markers [74]. Together with evidence on the intestinal barrier and microbiota, these findings indicate that assessment of TiO_2_ NPs should not be restricted to acute particle toxicity.

For a biofunctionalized TiO_2_ NP formulation, the critical question therefore becomes whether the initially observed advantage persists after remodeling of the biointerface and during repeated exposure. A reduction in nonspecific interactions in vitro matters only if it is not later offset by improved dispersion, increased biological accessibility, or greater particle persistence within biological compartments.

Thus, the criterion for successful biofunctionalization differs fundamentally between the two materials: for ZnO NPs, it requires simultaneous control of interactions involving the particulate fraction and Zn^2+^ release, whereas for TiO_2_ NPs, it depends predominantly on biointerface stability and limiting biological exposure to a persistent particle.

From a biofunctionalization perspective, these findings change the criteria for defining “safety” in the context of oral exposure. It is not sufficient for a biofunctionalized nanoparticle to exhibit lower cytotoxicity than its non-functionalized counterpart under the initial testing conditions. The advantage must persist after digestion, must not be achieved at the cost of increased exposure of the microbiota or intestinal epithelium, must limit systemic effects, and must remain compatible with material-specific mechanisms-dissolution and Zn^2+^ release for ZnO NPs and persistence and surface interactions for TiO_2_ NPs. Oral safety therefore tests the robustness of the engineered biointerface and the persistence of the toxicological advantage under realistic exposure conditions, rather than merely an additional toxicity endpoint.

## 9. A Safe-by-Design Framework for the Biofunctionalization of ZnO and TiO_2_ Nanoparticles

The safe-by-design principle requires integrating safety into the design of a nanoformulation from the stage at which its intended function is defined, rather than assessing it only after a material with the desired technological performance has been developed. For biofunctionalized ZnO NPs and TiO_2_ NPs, this approach matters because, as the evidence discussed in the preceding sections shows, modifying the biointerface does not predictably result in detoxification. It may reduce nonspecific interactions, but it may also increase stability, biological accessibility, or cellular uptake, or preserve and redirect nanoparticle reactivity toward specific targets. Consequently, a safe design must simultaneously consider the intrinsic hazard of the nanoparticle core, the function of the biointerface, material behavior in the exposure environment, and the final toxicological outcome.

Start with a clear definition of the intended function and exposure scenario. A nanoparticle intended for antimicrobial applications, UV protection, food-related applications, or delivery of an active molecule cannot be optimized according to the same toxicological criteria. At the same time, the design must match the material’s dominant mechanism. For ZnO NPs, dissolution and Zn^2+^ release must be evaluated together with effects specific to the particulate fraction, because reducing direct particle-cell contact does not guarantee elimination of toxicity following subsequent dissolution [11]. For TiO_2_ NPs, which are considerably more persistent, controlling surface reactivity, aggregation, photoreactivity, and biological interactions becomes a priority. This fundamental difference means no universal “safer” biofunctionalization strategy can apply to both materials. Design criteria must therefore be defined based on each nanomaterial’s dominant hazard mechanism.

At the next stage, the biointerface must be selected based on the function it performs within the nano-bio system, rather than solely on the biocompatibility of the biomolecule used. A protein, polysaccharide, peptide, or biosurfactant may simultaneously modify surface charge, colloidal stability, protein adsorption, membrane accessibility, cellular uptake, and surface reactivity. For this reason, ligand biocompatibility does not guarantee the safety of the resulting nanoformulation. The comparative studies discussed above illustrate this variability precisely: chitosan and alginate produced different toxicological effects on ZnO NPs, whereas association of TiO_2_ NPs with food proteins increased stability and cellular accessibility [17,18]. For applications involving intentional biological activity, the objective is therefore not the global elimination of reactivity but optimization of toxicological selectivity-limiting effects on non-target systems while preserving or enhancing the intended function.

Characterization of the material immediately after synthesis, however, represents only the beginning of the assessment. Following contact with proteins, electrolytes, food matrices, or gastrointestinal fluids, the engineered biointerface may become masked, exchanged, or reorganized, while aggregation, dissolution, and the biologically accessible dose may change substantially [21,49,50]. Physicochemical characterization and toxicological assessment should therefore be integrated within the same experimental workflow and conducted under conditions that approximate the intended exposure scenario as closely as possible. The material requiring toxicological evaluation is not merely the initially biofunctionalized nanoparticle but its effective form after transformation within the exposure environment.

This principle also changes how experimental comparisons should be designed. Demonstrating that biofunctionalization produces a toxicological advantage requires controls that allow causal attribution: the same nanoparticle core with and without biofunctionalization, the free biomolecule, and, where relevant, the linker or matrix used. For ZnO NPs, an ionic comparator is required when Zn^2+^ release may contribute to the observed effect. This requirement becomes even more important for biogenic or multicomponent systems, in which changes in particle size, morphology, structural defects, or surface composition may accompany biofunctionalization. Without such comparators, lower toxicity cannot be confidently attributed to the biointerface. An appropriate experimental design must therefore distinguish the effect of the biointerface from those of the nanoparticle core, the free biomolecule, and structural transformations associated with functionalization.

**Figure 3:**
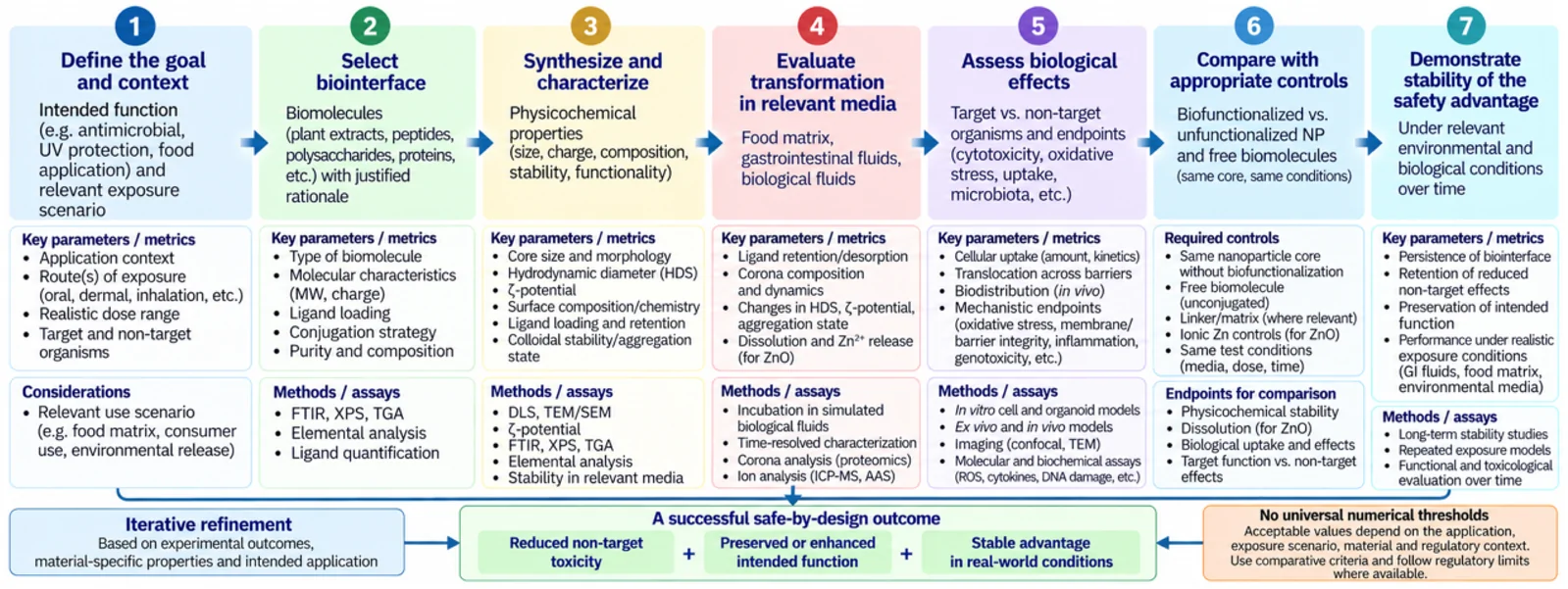
Proposed safe-by-design framework for biofunctionalized ZnO and TiO_2_ nanoparticles.

Operationally, each step of this framework should be linked to measurable parameters and appropriate controls. Initial characterization should include core size and morphology, hydrodynamic diameter, ζ-potential, aggregation state, surface composition, and ligand loading or retention. Exposure-relevant transformation should be assessed through time-resolved changes in these parameters, together with corona remodeling and, for ZnO, dissolution and Zn^2+^ release. Biological assessment should combine effective exposure or cellular uptake with mechanistic endpoints such as oxidative stress, membrane or barrier integrity, inflammatory responses, and other application-relevant effects. Comparisons should include the same nanoparticle core with and without biofunctionalization, the free biomolecule and, where relevant, linker/matrix and ionic Zn controls. The final decision should integrate three dimensions: reduction of non-target effects, preservation of the intended function, and persistence of this advantage after exposure-relevant transformation. Universal numerical thresholds for these parameters cannot presently be defined, because an acceptable value depends on the intended application, exposure scenario, material-specific hazard mechanism, and relevant regulatory context. Accordingly, the framework uses comparative decision criteria rather than arbitrary universal cut-offs; where regulatory or application-specific thresholds exist, these should take precedence in the final safety assessment. Finally, a safe-by-design strategy should be demonstrated by the persistence of the toxicological advantage rather than by a favorable result in a single experimental system. A biofunctionalization strategy can be considered genuinely advantageous only if it simultaneously meets three conditions: (i) it reduces non-target toxicity, (ii) it preserves or improves the intended function, and (iii) it maintains this advantage after exposure-relevant transformations. These three criteria—safety, functionality, and robustness of the advantage following biointerface transformation—form the basis of the safe-by-design framework proposed in Figure 3.

### Regulatory and Ecotoxicological Considerations for Safe-by-Design Biofunctionalization

The proposed framework is conceptually consistent with current regulatory approaches that emphasize nanomaterial-specific physicochemical characterization, transformation, exposure, and biological effects, although the scope and requirements differ among regulatory authorities. EFSA guidance for food and feed applications specifically addresses the identification and characterization of small particles, including nanoparticles, and the need to consider particle-related properties in safety assessment [75]. ECHA similarly incorporates nanoform-specific physicochemical, fate, exposure, and hazard considerations within the REACH framework [76]. The FDA applies a product-specific, case-by-case approach and considers whether nanoscale dimensions or related properties may affect the identity, safety, effectiveness, or regulatory status of a product [77]. Thus, the present framework should be regarded as complementary to, rather than a substitute for, regulatory assessment: its specific contribution is to emphasize whether the engineered biointerface and the associated toxicological advantage remain stable throughout the relevant exposure pathway.

Environmental safety represents an additional dimension of this assessment. Experimental evidence shows that both ZnO and TiO_2_ NPs can undergo bioaccumulation and trophic transfer, but the extent of these processes depends on particle properties, exposure conditions, and food-web structure [78,79]. For TiO_2_, particle size and crystalline composition influence bioconcentration and biomagnification [80], while trophic transfer does not necessarily result in biomagnification [81]. These distinctions are also material-specific: the relative persistence of TiO_2_ increases the relevance of particle retention and trophic transfer, whereas ZnO fate must additionally be interpreted in relation to dissolution and Zn^2+^ release. Importantly, comparable information for biofunctionalized ZnO and TiO_2_ remains scarce. Future safe-by-design assessment should therefore determine whether biofunctionalization modifies environmental persistence, bioaccumulation, and trophic transfer rather than assuming that reduced toxicity in cellular models translates into reduced environmental risk.

## 10. Knowledge Gaps and Research Priorities

The safe-by-design framework proposed in Section 9 defines the type of evidence required to demonstrate a robust toxicological advantage. However, the available literature indicates that this evidence is still insufficient. The main discrepancies between the requirements of the proposed framework and the current experimental basis are summarized in Table 3.

## 11. Conclusions

Biofunctionalization of ZnO and TiO_2_ nanoparticles enables control over the interface between the inorganic core and the biological environment. Still, the available evidence does not support it as an intrinsically or universally effective detoxification strategy. Depending on the biomolecule, surface architecture, nanoparticle properties, and exposure context, biofunctionalization may reduce toxicity, produce only minor changes, increase biological accessibility, or redirect the effect towards specific biological targets.

Comparative studies therefore suggest that biofunctionalization reprograms nanoparticle-biological system interactions. Changes in surface charge, aggregation, surface accessibility, and cellular uptake can modify the biologically effective dose and the localization of the effects. In some systems, core reactivity is reduced, whereas in others it is preserved but becomes more selective towards specific biological targets. From this perspective, reducing non-target effects while preserving the intended biological function offers a more informative safety criterion than a global reduction in cytotoxicity.

However, the identity conferred by biofunctionalization is not permanent. Contact with food components and gastrointestinal fluids may remodel the engineered surface through biomolecule adsorption, biointerface reorganization, and changes in aggregation state; for ZnO NPs, dissolution and Zn^2+^ release add another transformation pathway. For TiO_2_ NPs, the particles’ relative persistence shifts the emphasis to biointerface stability and control of surface interactions throughout exposure. “Toxicological identity” should therefore be understood as an emergent property arising from the interplay between the initial physicochemical identity, the engineered biointerface, transformations occurring along the exposure pathway, and accessibility to biological targets.

This dynamic behavior is particularly important in the context of oral exposure, for which direct comparative evidence on biofunctionalized ZnO and TiO_2_ nanoparticles remains insufficient. An advantage demonstrated in simplified experimental systems cannot automatically be extrapolated to ingestion without demonstrating that it persists following interactions with the food matrix and gastrointestinal transformations.

Although this review focuses on ZnO and TiO_2_, some of the broader principles identified here may extend to other metal oxide nanoparticles, particularly those related to biointerface remodeling, aggregation-dependent changes in effective exposure, and the need for appropriate comparative controls. However, material-specific conclusions should not be extrapolated without experimental validation, because dissolution, ion release, redox activity, surface reactivity, and persistence differ among metal oxides. Thus, the conceptual framework may be transferable, whereas the specific toxicological outcomes identified for ZnO and TiO_2_ are not necessarily generalizable to other metal oxides.

Overall, biofunctionalization should be regarded as a tool for controlling the biointerface and, consequently, the toxicological identity of nanoparticles, rather than as a guarantee of safety. It can make ZnO and TiO_2_ nanoparticles safer only when surface modification produces a demonstrable, predictable, and sufficiently robust toxicological advantage that persists after biointerface transformation under realistic exposure conditions. From this perspective, designing a safer nanoparticle involves not merely reducing its initial reactivity but controlling how function, biointerface, and hazard evolve together throughout biological exposure.

## Figures and Tables

**Table 1:** Mechanistic differences between ZnO and TiO_2_ nanoparticles relevant to biofunctionalization and toxicological identity.

Feature	ZnO Nanoparticles	TiO_2_ Nanoparticles	Implication for Biofunctionalization	Toxicological Relevance	Representative References
Dissolution, ionic contribution and particulate persistence	Dissolution is strongly environment-dependent and increases markedly under acidic and intracellular conditions. Zn^2+^ release can substantially contribute to toxicity, although particle-specific effects remain. Persistence of the original particulate form is therefore variable.	Dissolution is generally very low under biologically relevant conditions; TiO_2_ is comparatively biodurable and persists predominantly in particulate form.	For ZnO, the biointerface may modify dissolution kinetics but cannot be assumed to prevent dissolution. For TiO_2_, modulation of the persistent particulate interface is more important.	ZnO assessment should distinguish particulate from ionic effects, whereas TiO_2_ toxicity is more strongly associated with persistent particle-surface interactions than with dissolution-mediated effects.	[11,32]
Aggregation and colloidal stability	Strongly medium-dependent and coupled to surface chemistry, biomolecule adsorption and dissolution.	Strongly medium-dependent and a major determinant of the effective particulate exposure. Protein association or surface modification can markedly alter dispersion.	Biofunctionalization can stabilize or destabilize dispersions depending on ligand chemistry, charge and medium composition.	Greater colloidal stability does not necessarily mean lower toxicity; improved dispersion may increase biological accessibility and effective dose.	[18,31,40]
Surface charge and interfacial chemistry	Surface charge affects aggregation, biomolecule adsorption, membrane interactions and accessibility of the ZnO surface; these effects coexist with dissolution-related transformations.	Surface charge and surface chemistry strongly influence aggregation, protein adsorption, membrane association and cellular uptake.	Biofunctionalization can markedly change ζ-potential, hydrophilicity and availability of reactive surface sites.	Changes in interfacial properties may reduce nonspecific interactions or, conversely, promote interaction with selected biological targets.	[14,25,32,34,46]
Biomolecular corona and biointerface evolution	Proteins and other biomolecules can modify dispersion, surface properties and biological recognition; corona evolution may occur simultaneously with ZnO dissolution.	Particularly important because the persistent TiO_2_ surface remains available for biomolecule adsorption and corona reorganization. Corona composition is strongly influenced by medium and exposure history.	The deliberately designed biointerface may be masked, exchanged or reorganized after contact with biological or food matrices.	The biologically effective surface may differ substantially from the initially characterized formulation; therefore, initial biofunctionalization does not necessarily predict final toxicological identity.	[21,47,48,49,50]
Surface reactivity, ROS generation and photoreactivity	ROS-related effects can coexist with dissolution-mediated toxicity; the relative contribution depends on particle properties and exposure conditions.	Surface-mediated ROS generation is a major mechanism, particularly for photoactive TiO_2_; photocatalytic activity may persist after incorporation into biological/polymeric matrices.	Biofunctionalization may shield reactive sites, attenuate ROS formation, preserve functional reactivity or redirect it toward selected targets.	Reduced global ROS generation is not necessarily the only desirable outcome: preservation of target-specific reactivity can improve the safety/function balance.	[14,25,33,34,36,45]
Cellular uptake and biological accessibility	Determined jointly by aggregation, surface charge, corona formation, dissolution and transformation during exposure.	Strongly affected by dispersion state, corona and targeting ligands; improved colloidal stability can increase penetration or uptake.	Biofunctionalization may decrease nonspecific uptake but can also intentionally or unintentionally increase biological accessibility.	Lower intrinsic surface reactivity can coexist with a higher biologically effective exposure; uptake therefore needs to be interpreted together with hazard.	[15,18,19,32]
Transformation in food and gastrointestinal environments	Sequential changes in pH can promote dissolution, aggregation, biomolecular interactions and potentially secondary transformations; food components can modify these processes.	Transformation is dominated more by aggregation, dynamic protein/lipid corona formation, food-matrix interactions and persistence of the particulate form.	The stability and identity of the designed biointerface should be evaluated after sequential exposure to food matrices and gastrointestinal fluids rather than only in the starting formulation.	Safety demonstrated for the initial formulation cannot be directly extrapolated to the material reaching the intestinal epithelium.	[21,51,52]
Priority parameters for exposure-relevant characterization	Particle state and size distribution, dissolution/Zn^2+^ release, aggregation, surface/biointerface stability, corona evolution and effective dose.	Particle state and size distribution, surface composition/reactivity, aggregation, corona evolution, photoreactivity, uptake and particulate persistence.	Characterization should be material-specific, time-resolved and performed in exposure-relevant media, ideally before and after biofunctionalization and during biological transformation.	A generic characterization scheme is insufficient to predict the toxicological identity of both oxides or the safety advantage produced by biofunctionalization.	[11,14,18,21]

**Table 2:** Comparative evidence on how deliberate biofunctionalization modifies the toxicological identity of ZnO and TiO_2_ nanoparticles.

Study	NP/Biofunctionalization and Comparator	Main Physicochemical Effect	Biological Evidence/Outcome	Toxicological Interpretation
Vian et al., 2021 [18]	TiO_2_ with adsorbed gelatin or β-lactoglobulin; comparator: Uncoated TiO_2_.	Protein adsorption altered colloidal behavior and reduced aggregation/improved stability in biological media.	HT-29 intestinal cells; cellular penetration. Outcome: Increased biological access/uptake.	Protein coating improved dispersion and promoted cellular interaction and penetration rather than simply reducing biological exposure.
Caselli, et al., 2024 [25]	TiO_2_ coated with LL-37; comparator: Bare TiO_2_.	ζ-potential became strongly positive and colloidal stability increased; coating did not markedly suppress ROS generation.	Bacteria-like vs. mammalian-like lipid membranes; bacteria; human monocytes. Outcome: Altered selectivity.	LL-37 increased binding to anionic bacterial-like membranes while preserving photocatalytic ROS generation, thereby redirecting photocatalytic activity preferentially toward bacterial targets.
Caselli, et al., 2024 [33]	TiO_2_ coated with KYE21 or WWWKYE21 peptides; comparator: Bare TiO_2_.	Peptide coatings retained ROS generation; W-tagging increased peptide hydrophobicity and binding to bacteria-like membranes while preserving photocatalytic activity.	Bacteria-like and mammalian-like membranes; microorganisms/cells. Outcome: Altered selectivity; increased targeted activity.	The coating did not deactivate TiO_2_; instead, it increased binding to anionic bacterial membranes and localized photocatalytic damage.
Caselli, et al., 2024 [34]	TiO_2_ coated with LL-37; comparator: Bare TiO_2_.	Positive peptide coating retained ROS generation and markedly increased association with LPS and LTA.	LPS, LTA; THP1 human monocytes; NF-κB/AP-1 and LDH. Outcome: Altered selectivity; enhanced suppression of LPS/LTA-induced inflammatory activation with low host-cell toxicity.	LL-37 redirected photocatalytic reactivity toward bacterial surface molecules, enhancing their binding, degradation and capture while maintaining low monocyte toxicity.
Cesa et al., 2026 [14]	TiO_2_ P25 coated with sodium surfactin; comparator: Pristine P25; surfactin alone; physical mixture.	Surface charge and dispersion changed; visible-light photoreactivity was strongly suppressed.	BEAS-2B, RAW264.7 and HSF cells; algae; zebrafish embryos. Outcome: Reduced toxicity overall, but context-dependent.	Surfactin coating reduced photoreactivity/ROS-related effects and lowered toxicity in several models, although high-dose macrophage responses were less favorable.
Karthikeyan et al., 2021 [17]	ZnO hybridized with chitosan or alginate; comparator: Bare ZnO.	Biopolymers altered particle size, aggregation and surface structure while retaining wurtzite ZnO.	HepG2 cells; cytotoxicity. Outcome: Coating-dependent: chitosan reduced toxicity; alginate approximately like bare ZnO.	Different biomolecular coatings applied to the same oxide produced different toxicological outcomes, showing that coating identity is critical.
Jose et al., 2024 [15]	TiO_2_ modified with BSA -PyBA; comparator: Unmodified TiO_2_.	Biomolecular surface layer altered surface charge/stability.	Normal rat spleen cells and Dalton’s lymphoma ascites (DLA) cancer cells; cytotoxicity. Outcome: Increased cytotoxicity, preferentially toward tumor cells	The modified system increased cytotoxicity preferentially toward DLA tumor cells, indicating enhanced tumor selectivity rather than generalized detoxification.
Jana et al., 2022 [16]	ZnO encapsulated in functionalized chitosan derivatives; comparator: Bare ZnO.	Polymer encapsulation modified the ZnO nano-interface.	Vero cells/human cytomegalovirus; cytotoxicity and antiviral activity. Outcome: Reduced host-cell toxicity with largely retained antiviral efficacy.	Functionalized chitosan-ZnO retained substantial antiviral activity while reducing host-cell cytotoxicity, thereby improving the efficacy-to-toxicity balance.
Dayan et al., 2018 [19]	TiO_2_ conjugated with DLDH-RGD protein construct; comparator: TiO_2_; TiO_2_-DLDH.	Stable coordinative protein binding; targeting motif introduced without preventing TiO_2_ binding.	B16F10 melanoma vs. HEK293 cells; Ultraviolet A-triggered cytotoxicity. Outcome: Strongly altered/selective toxicity.	RGD-mediated integrin targeting spatially concentrated TiO_2_ phototoxicity in integrin-positive cancer cells while preserving only baseline TiO_2_ effects in low-integrin normal cells.
Basak et al., 2014 [20]	ZnO biofunctionalized with acidic diacetate sophorolipid; comparator: Naked ZnO	Similar crystallite size but altered/smoother surface morphology after biofunctionalization.	*Salmonella enterica* and *Candida albicans*; growth, protein leakage, lactate dehydrogenase and morphology. Outcome: Enhanced antimicrobial potency toward microbial targets.	Sophorolipid functionalization enhanced NP-cell interaction and membrane damage, increasing antimicrobial activity relative to naked ZnO.

**Table 3:** Key knowledge gaps and research priorities for evaluating the safety of biofunctionalized ZnO and TiO_2_ nanoparticles.

Current Knowledge Gap	Why It Matters	Evidence Required	Research Priority
Limited causal attribution of biofunctionalization effects	Many studies simultaneously change synthesis route, size, morphology, defect structure and surface composition; improved safety therefore cannot be assigned specifically to the biointerface.	Matched comparisons using the same nanoparticle core with and without biofunctionalization; free biomolecule and matrix/linker controls; for ZnO, an appropriate ionic Zn control.	Establish causal relationships between the designed biointerface and toxicological outcome.
Biointerface characterized mainly before biological exposure	The designed surface may be masked, exchanged or reorganized by proteins, lipids, electrolytes and food/biological matrices.	Time- and environment-resolved measurements of surface composition, hydrodynamic size, ζ-potential, aggregation, ligand retention/exchange and corona evolution.	Characterize the dynamic biointerface under the conditions in which toxicity occurs.
Insufficient distinction between ZnO- and TiO_2_-specific pathways	ZnO combines particulate effects with environment-dependent Zn^2+^ release, whereas TiO_2_ is more persistent and its toxicological behavior is more strongly governed by surface reactivity, aggregation, corona formation and photoreactivity.	ZnO: time-resolved dissolution, particulate fraction and Zn^2+^ measured together, using matched non-biofunctionalized and ionic controls. TiO_2_: persistence, surface reactivity, aggregation, corona and photoreactivity.	Develop material-specific testing strategies rather than treating metal-oxide nanoparticles as a homogeneous class.
Scarce comparative evidence after oral exposure	Safety advantages observed in simple in vitro systems may not survive food-matrix, gastrointestinal, microbiota and intestinal-barrier transformations.	Direct biofunctionalized-versus-pristine comparisons across a tiered oral-exposure sequence comprising simulated digestion, intestinal in vitro and ex vivo models, and targeted in vivo validation, including microbiota, barrier integrity, absorption, and biodistribution; selected co-exposure scenarios with relevant food additives, contaminants, or pharmaceuticals.	Determine whether the safety advantage survives gastrointestinal transformation and oral exposure.
Reliance on nominal dose and single toxicity endpoints	Biofunctionalization changes dispersion, sedimentation, dissolution and uptake, so equal nominal concentrations may produce different effective exposures; single viability endpoints can miss selectivity.	Quantification of administered, dispersed, sedimented, dissolved and internalized fractions combined with mechanistic and target-versus non-target endpoints.	Link effective dose to multidimensional response and characterize target versus non-target selectivity.
Limited evidence that the safety advantage is persistent and predictive	A formulation may appear safer initially but lose that advantage after corona formation, ligand exchange, digestion, pH changes or biodistribution.	Longitudinal designs following the same formulation from initial characterization through transformation, target interaction and final toxicological outcome in progressively complex models.	Demonstrate persistence and predictability of the safety advantage in the intended exposure scenario.
Limited environmental fate data for biofunctionalized NPs	Biofunctionalization may modify persistence, dissolution, organism uptake and trophic transfer.	Comparative pristine-versus-biofunctionalized studies addressing persistence, bioaccumulation and trophic transfer under environmentally relevant conditions.	Determine whether biofunctionalization reduces, increases, or redirects environmental exposure and risk

## Data Availability

No new data were created or analyzed in this study. Data sharing is not applicable to this article.

## References

[1] Dayan A., Fleminger G. (2026). Titanium Dioxide in Biomedical and Environmental Nanotechnology: From Photocatalytic Detoxification to Targeted Therapeutics. Molecules.

[2] Huq M.A., Apu M.A.I., Ashrafudoulla M., Rahman M.M., Parvez M.A.K., Balusamy S.R., Akter S., Rahman M.S. (2023). Bioactive ZnO Nanoparticles: Biosynthesis, Characterization and Potential Antimicrobial Applications. Pharmaceutics.

[3] Rani S., Kumar P., Dahiya P., Dang A.S., Suneja P. (2022). Biogenic Synthesis of Zinc Nanoparticles, Their Applications, and Toxicity Prospects. Front. Microbiol..

[4] Sportelli M.C., Gaudiuso C., Volpe A., Izzi M., Picca R.A., Ancona A., Cioffi N. (2022). Biogenic Synthesis of ZnO Nanoparticles and Their Application as Bioactive Agents: A Critical Overview. Reactions.

[5] Rahman A., Harunsani M.H., Tan A.L., Khan M.M. (2021). Zinc Oxide and Zinc Oxide-Based Nanostructures: Biogenic and Phytogenic Synthesis, Properties and Applications. Bioprocess Biosyst. Eng..

[6] Singh R., Srinivas S.P., Kumawat M., Daima H.K. (2024). Ligand-Based Surface Engineering of Nanomaterials: Trends, Challenges, and Biomedical Perspectives. OpenNano.

[7] Nitschke M., Marangon C.A. (2022). Microbial Surfactants in Nanotechnology: Recent Trends and Applications. Crit. Rev. Biotechnol..

[8] Sampath S., Madhavan Y., Muralidharan M., Sunderam V., Lawrance A.V., Muthupandian S. (2022). A Review on Algal Mediated Synthesis of Metal and Metal Oxide Nanoparticles and Their Emerging Biomedical Potential. J. Biotechnol..

[9] Pavan C., Santalucia R., Escolano-Casado G., Ugliengo P., Mino L., Turci F. (2023). Physico-Chemical Approaches to Investigate Surface Hydroxyls as Determinants of Molecular Initiating Events in Oxide Particle Toxicity. Int. J. Mol. Sci..

[10] Neetika S.M., Thakur P., Gaur P., Rani G.M., Rustagi S., Talreja R.K., Chaudhary V. (2023). Cancer Treatment and Toxicity Outlook of Nanoparticles. Environ. Res..

[11] Guo Z., Luo Y., Zhang P., Chetwynd A.J., Qunhui Xie H., Abdolahpur Monikh F., Tao W., Xie C., Liu Y., Xu L. (2020). Deciphering the Particle Specific Effects on Metabolism in Rat Liver and Plasma from ZnO Nanoparticles versus Ionic Zn Exposure. Environ. Int..

[12] Kopac T. (2021). Protein Corona, Understanding the Nanoparticle-Protein Interactions and Future Perspectives: A Critical Review. Int. J. Biol. Macromol..

[13] Wheeler K.E., Chetwynd A.J., Fahy K.M., Hong B.S., Tochihuitl J.A., Foster L.A., Lynch I. (2021). Environmental Dimensions of the Protein Corona. Nat. Nanotechnol..

[14] Cesa E., Costa A.L., Faccani L., Fornara D., Yilmaz H., Culha M., Adiguzel S., Sener G., Cicek N., Okyay T.M. (2026). Validation of a Lipopeptide Approach to a Safe-and-Sustainable-by-Design Strategy on TiO_2_ Nanoparticles UV Filters. J. Colloid Interface Sci..

[15] Jose D.E., Mathew T.V. (2024). Surface Modification of Titania Nanoparticles with BSA Engineered Coating: Investigation of Antimicrobial and Anticancer Activities. Inorg. Chem. Commun..

[16] Jana B., Chatterjee A., Roy D., Ghorai S., Pan D., Pramanik S.K., Chakraborty N., Ganguly J. (2022). Chitosan/Benzyloxy-Benzaldehyde Modified ZnO Nano Template Having Optimized and Distinct Antiviral Potency to Human Cytomegalovirus. Carbohydr. Polym..

[17] Karthikeyan C., Tharmalingam N., Varaprasad K., Mylonakis E., Yallapu M.M. (2021). Biocidal and Biocompatible Hybrid Nanomaterials from Biomolecule Chitosan, Alginate and ZnO. Carbohydr. Polym..

[18] Vian R., Salehi H., Lapierre M., Cuisinier F., Cavaillès V., Balme S. (2021). Adsorption of Proteins on TiO_2_ Particles Influences Their Aggregation and Cell Penetration. Food Chem..

[19] Dayan A., Fleminger G., Ashur-Fabian O. (2018). RGD-Modified Dihydrolipoamide Dehydrogenase Conjugated to Titanium Dioxide Nanoparticles—Switchable Integrin-Targeted Photodynamic Treatment of Melanoma Cells. RSC Adv..

[20] Basak G., Das D., Das N. (2014). Dual Role of Acidic Diacetate Sophorolipid as Biostabilizer for ZnO Nanoparticle Synthesis and Biofunctionalizing Agent against Salmonella enterica and Candida albicans. J. Microbiol. Biotechnol..

[21] Coreas R., Cao X., Deloid G.M., Demokritou P., Zhong W. (2020). Lipid and Protein Corona of Food-Grade TiO_2_ Nanoparticles in Simulated Gastrointestinal Digestion. NanoImpact.

[22] El-Belely E.F., Farag M.M.S., Said H.A., Amin A.S., Azab E., Gobouri A.A., Fouda A. (2021). Green Synthesis of Zinc Oxide Nanoparticles (ZnO-NPs) Using *Arthrospira platensis* (Class: Cyanophyceae) and Evaluation of Their Biomedical Activities. Nanomaterials.

[23] Karthikeyan C., Varaprasad K., Akbari-Fakhrabadi A., Hameed A.S.H., Sadiku R. (2020). Biomolecule Chitosan, Curcumin and ZnO-Based Antibacterial Nanomaterial, via a One-Pot Process. Carbohydr. Polym..

[24] Sarkar S., Debnath S.K., Srivastava R., Kulkarni A.R. (2022). Continuous Flow Scale-up of Biofunctionalized Defective ZnO Quantum Dots: A Safer Inorganic Ingredient for Skin UV Protection. Acta Biomater..

[25] Caselli L., Parra-Ortiz E., Micciulla S., Skoda M.W.A., Häffner S.M., Nielsen E.M., van der Plas M.J.A., Malmsten M. (2024). Boosting Membrane Interactions and Antimicrobial Effects of Photocatalytic Titanium Dioxide Nanoparticles by Peptide Coating. Small.

[26] Rahujo S., Talpur F.N., Laghari A.Q., Mert O., Afridi H.I., Ali Z., Razzaque N., Chandio I., Zaman N., Abro M. (2026). Biofunctionalized Titanium Dioxide Nanoparticles with Bovine Serum Albumin for Targeted Cytotoxicity in MCF-7 Breast Cancer Cells. Indones. J. Chem..

[27] Niu B., Qiao S., Sun Y., Niu Y. (2025). Enhancing Dispersion Stability of Nano Zinc Oxide with Rhamnolipids and Evaluating Antibacterial Activity against Harmful Corn Fungi. Front. Microbiol..

[28] Jadhao A.D., Shende S., Ingle P., Gade A., Hajare S.W., Ingole R.S. (2020). Biogenic Synthesis of Zinc Oxide Nanoparticles by Bryophyllum pinnatum and Its Acute Oral Toxicity Evaluation in Wistar Rats. IEEE Trans. Nanobioscience.

[29] Abinaya M., Vaseeharan B., Divya M., Sharmili A., Govindarajan M., Alharbi N.S., Kadaikunnan S., Khaled J.M., Benelli G. (2018). Bacterial Exopolysaccharide (EPS)-Coated ZnO Nanoparticles Showed High Antibiofilm Activity and Larvicidal Toxicity against Malaria and Zika Virus Vectors. J. Trace Elem. Med. Biol..

[30] Fouda A., EL-Din Hassan S., Salem S.S., Shaheen T.I. (2018). In-Vitro Cytotoxicity, Antibacterial, and UV Protection Properties of the Biosynthesized Zinc Oxide Nanoparticles for Medical Textile Applications. Microb. Pathog..

[31] Mohammad F., Bwatanglang I.B., Al-Lohedan H.A., Shaik J.P., Al-Tilasi H.H., Soleiman A.A. (2023). Influence of Surface Coating Towards the Controlled Toxicity of ZnO Nanoparticles In Vitro. Coatings.

[32] Luo M., Shen C., Feltis B.N., Martin L.L., Hughes A.E., Wright P.F., Turney T.W. (2014). Reducing ZnO Nanoparticle Cytotoxicity by Surface Modification. Nanoscale.

[33] Caselli L., Traini T., Micciulla S., Sebastiani F., Köhler S., Nielsen E.M., Diedrichsen R.G., Skoda M.W.A., Malmsten M. (2024). Antimicrobial Peptide Coating of TiO_2_ Nanoparticles for Boosted Antimicrobial Effects. Adv. Funct. Mater..

[34] Caselli L., Du G., Micciulla S., Traini T., Sebastiani F., Diedrichsen R.G., Köhler S., Skoda M.W.A., van der Plas M.J.A., Malmsten M. (2024). Photocatalytic Degradation of Bacterial Lipopolysaccharides by Peptide-Coated TiO_2_ Nanoparticles. ACS Appl. Mater. Interfaces.

[35] Karthikeyan C., Jayaramudu T., Núñez D., Jara N., Opazo-Capurro A., Varaprasad K., Kim K., Yallapu M.M., Sadiku R. (2023). Hybrid Nanomaterial Composed of Chitosan, Curcumin, ZnO and TiO_2_ for Antibacterial Therapies. Int. J. Biol. Macromol..

[36] Samal P., Satpathy S., Rath G., Sabut T., Sahoo A.-P., Arakha M. (2026). Lignin-Encapsulated ZnO Nano-Hybrids Exhibit Enhanced Antibacterial and Photocatalytic Activity via ROS-Mediated Oxidative Stress and Bacterial Membrane Disruption. ACS Omega.

[37] Mandal S., Hazra C., Joshi N., Sen R., Das S., Das K. (2025). Evaluating Antimicrobial Activity: Biogenic vs. Chemically Functionalized Zinc Oxide Nanoparticles. Mater. Lett..

[38] Ghaffari S.B., Sarrafzadeh M.H., Fakhroueian Z., Shahriari S., Khorramizadeh M.R. (2017). Functionalization of ZnO Nanoparticles by 3-Mercaptopropionic Acid for Aqueous Curcumin Delivery: Synthesis, Characterization, and Anticancer Assessment. Mater. Sci. Eng. C.

[39] Zheng X., Qian C., Wang H., Bi J., Qi X., Shen S., Cao J. (2025). Phycocyanin- and Polydopamine-Conjugated Titania Nanoparticles for UV Protection. ACS Appl. Nano Mater..

[40] Josiah A.J., Pillai S.K., Cordier W., Nell M., Lall N., Twilley D., Ray S.S. (2024). One-Step Synthesis of Metal Oxide Nanoparticles Using Cannabidiol: Characterisation and Cytotoxicity Assessment in Human Keratinocyte Cells. ChemistrySelect.

[41] Yu Q., Wang H., Peng Q., Li Y., Liu Z., Li M. (2017). Different Toxicity of Anatase and Rutile TiO_2_ Nanoparticles on Macrophages: Involvement of Differences in Affinity to Proteins and Phospholipids. J. Hazard. Mater..

[42] Gerloff K., Fenoglio I., Carella E., Kolling J., Albrecht C., Boots A.W., Förster I., Schins R.P. (2012). Distinctive Toxicity of TiO_2_ Rutile/Anatase Mixed Phase Nanoparticles on Caco-2 Cells. Chem. Res. Toxicol..

[43] Fresegna A.M., Ursini C.L., Ciervo A., Maiello R., Casciardi S., Iavicoli S., Cavallo D. (2021). Assessment of the Influence of Crystalline Form on Cyto-Genotoxic and Inflammatory Effects Induced by TiO_2_ Nanoparticles on Human Bronchial and Alveolar Cells. Nanomaterials.

[44] Kunc F., Du X., Zborowski A., Johnston L.J., Kennedy D.C. (2025). Titanium Dioxide Nanoparticles—Physicochemical Characterization and Cytotoxic Risk. NanoImpact.

[45] Ferrara V., Marchetti M., Alfieri D., Targetti L., Scopelliti M., Pignataro B., Pavone F., Vetri V., Sancataldo G. (2023). Blue Light Activated Photodegradation of Biomacromolecules by N-Doped Titanium Dioxide in a Chitosan Hydrogel Matrix. J. Photochem. Photobiol. A Chem..

[46] Svensson F.G., Greijer B.H., Guerin T., Agback T., Agback P., Kessler V.G. (2026). Molecular Mechanisms behind the Potential Genotoxicity of Metal Oxide Nanoparticles: Nucleoside Deglycosylation Pathway. Dalton Trans..

[47] Tsokolakyan A.S., Hayrapetyan V.A., Asatryan H.V., Simonyan A.S., Dejneka A., Yeranosyan M.A. (2026). Novel Observation of SEIRA and SERS in Aluminum-Doped ZnO-BSA Protein Corona Complexes. Opt. Mater..

[48] Romano P., Bellingeri A., Morelli M., Lucignano R., Paccagnini E., Venditti I., Carpentieri A., Lupetti P., Corsi I., Leone S. (2026). Linking Protein Corona Composition to Ecotoxicological Outcomes: A Comparative Study of Different Engineered Nanoparticles in Mediterranean Sea Urchin *Paracentrotus lividus*. Environ. Sci. Nano.

[49] Kim J., Doudrick K. (2019). Emerging Investigator Series: Protein Adsorption and Transformation on Catalytic and Food-Grade TiO_2_ Nanoparticles in the Presence of Dissolved Organic Carbon. Environ. Sci. Nano.

[50] Shakiba S., Hakimian A., Barco L.R., Louie S.M. (2018). Dynamic Intermolecular Interactions Control Adsorption from Mixtures of Natural Organic Matter and Protein onto Titanium Dioxide Nanoparticles. Environ. Sci. Technol..

[51] Vaccari F., Zhang L., Giuberti G., Grasso A., Bandini F., García-Pérez P., Copat C., Lucini L., Dall’Asta M., Ferrante M. (2023). The Impact of Metallic Nanoparticles on Gut Fermentation Processes: An Integrated Metabolomics and Metagenomics Approach Following an in Vitro Digestion and Fecal Fermentation Model. J. Hazard. Mater..

[52] Yan J., Chen Q., Tian L., Li K., Lai W., Bian L., Han J., Jia R., Liu X., Xi Z. (2022). Intestinal Toxicity of Micro- and Nano-Particles of Foodborne Titanium Dioxide in Juvenile Mice: Disorders of Gut Microbiota-Host Co-Metabolites and Intestinal Barrier Damage. Sci. Total Environ..

[53] Rajkumar K.S., Sivagaami P., Ramkumar A., Murugadas A., Srinivasan V., Arun S., Kumar P.S., Thirumurugan R. (2022). Bio-Functionalized Zinc Oxide Nanoparticles: Potential Toxicity Impact on Freshwater Fish *Cyprinus carpio*. Chemosphere.

[54] Zokalih A.H., El Hawary S., El Sayed A.M., El Raey M.A., Abdelhameed M.F., Raafat K.M. (2026). *Frankenia hirsuta*-Derived Biofunctionalized ZnO Nanoparticles Attenuate Inflammation-Associated Tissue Injury: Integrated Pharmacological Evaluation Supported by Network Pharmacology and Molecular Docking. Biomed. Pharmacother..

[55] Alijagic A., Benada O., Kofroňová O., Cigna D., Pinsino A. (2019). Sea Urchin Extracellular Proteins Design a Complex Protein Corona on Titanium Dioxide Nanoparticle Surface Influencing Immune Cell Behavior. Front. Immunol..

[56] Stauber R.H., Westmeier D., Wandrey M., Becker S., Docter D., Ding G.-B., Thines E., Knauer S.K., Siemer S. (2020). Mechanisms of Nanotoxicity-Biomolecule Coronas Protect Pathological Fungi Against Nanoparticle-Based Eradication. Nanotoxicology.

[57] Baimanov D., Wang J., Zhang J., Liu K., Cong Y., Shi X., Zhang X., Li Y., Li X., Qiao R. (2022). In Situ Analysis of Nanoparticle Soft Corona and Dynamic Evolution. Nat. Commun..

[58] Yu Y., Luan Y., Dai W. (2022). Dynamic Process, Mechanisms, Influencing Factors and Study Methods of Protein Corona Formation. Int. J. Biol. Macromol..

[59] Sun Y., Zhou Y., Rehman M., Wang Y.F., Guo S. (2024). Protein Corona of Nanoparticles: Isolation and Analysis. Chem. Bio Eng..

[60] Binjawhar D.N., Al-Warhi T., Siddiqui G.A., Khan A. (2023). Probing the Interaction of Zinc Oxide Nanorods with Human Serum Albumin: A Spectroscopic Approach. J. Biotechnol..

[61] Sebők-Nagy K., Kóta Z., Kincses A., Fazekas Á.F., Dér A., László Z., Páli T. (2023). Spin-Label Electron Paramagnetic Resonance Spectroscopy Reveals Effects of Wastewater Filter Membrane Coated with Titanium Dioxide Nanoparticles on Bovine Serum Albumin. Molecules.

[62] Udnoor A., Lokolkar M., Yallur B.C., Kale R., Kalasad M.N., Katrahalli U., Manjunatha D.H. (2023). Monitoring the Interactions between Bovine Serum Albumin and ZnO/Ag Nanoparticles by Spectroscopic Techniques. J. Biomol. Struct. Dyn..

[63] OECD (2026). Test No. 322: Determination of the Solubility and Dissolution Rate of Nanomaterials for Environmental Fate Assessment.

[64] OECD (2020). Guidance Document for the Testing of Dissolution and Dispersion Stability of Nanomaterials, and the Use of the Data for Further Environmental Testing and Assessment.

[65] Lei L., Wang A., Dong L., Wu T., He W., Yang Y., Li J., Bi X., Cai Y., Guan X. (2026). Food-Grade TiO_2_ Impairs Intestinal Mucus Barrier via Disrupting the Gut Microbiota-ILA-Mucin Sulfation Axis: Novel Insights and Dietary Intervention Strategies. J. Nanobiotechnol..

[66] Yi J., Li Y., Mai Q., Li Y., Lin Y., Weng X., Ai Z., Li M., Shang P., Iqbal M. (2022). Hepatotoxicity and the Role of the Gut-Liver Axis in Dogs after Oral Administration of Zinc Oxide Nanoparticles. Metallomics.

[67] Jung E.B., Yu J., Choi S.J. (2021). Interaction between ZnO Nanoparticles and Albumin and Its Effect on Cytotoxicity, Cellular Uptake, Intestinal Transport, Toxicokinetics, and Acute Oral Toxicity. Nanomaterials.

[68] Yan X., Xu X., Guo M., Wang S., Gao S., Zhu S., Rong R. (2017). Synergistic Toxicity of ZnO Nanoparticles and Dimethoate in Mice: Enhancing Their Biodistribution by Synergistic Binding of Serum Albumin and Dimethoate to ZnO Nanoparticles. Environ. Toxicol..

[69] Wang C., Zhou Z., He Y., Li J., Cao Y. (2024). Influences of TiO_2_ Nanoparticle and Fipronil Co-Exposure on Metabolite Profiles in Mouse Intestines. J. Appl. Toxicol..

[70] Wang C., Liu Y., He Y., Li J., Cao Y. (2024). Combined Effects of TiO_2_ Nanoparticle and Fipronil Co-Exposure on Microbiota in Mouse Intestine. Food Chem. Toxicol..

[71] Safwat G., Mohamed A.A., Mohamed H.R.H. (2022). Estimation of Genotoxicity, Apoptosis and Oxidative Stress Induction by TiO_2_ Nanoparticles and Acrylamide Subacute Oral Coadministration in Mice. Sci. Rep..

[72] Chen J., Zhang S., Chen C., Jiang X., Qiu J., Qiu Y., Zhang Y., Wang T., Qin X., Zou Z. (2020). Crosstalk of Gut Microbiota and Serum/Hippocampus Metabolites in Neurobehavioral Impairments Induced by Zinc Oxide Nanoparticles. Nanoscale.

[73] Duan Y., Yang Y., Zhang Z., Xing Y., Li H. (2023). Toxicity of Titanium Dioxide Nanoparticles on the Histology, Liver Physiological and Metabolism, and Intestinal Microbiota of Grouper. Mar. Pollut. Bull..

[74] Chen Z., Han S., Zheng P., Zhou D., Zhou S., Jia G. (2020). Effect of Oral Exposure to Titanium Dioxide Nanoparticles on Lipid Metabolism in Sprague-Dawley Rats. Nanoscale.

[75] More S., Bampidis V., Benford D., Bragard C., Halldorsson T., Hernández-Jerez A., Bennekou S.H., Koutsoumanis K., Lambré C., EFSA Scientific Committee (2021). Guidance on Technical Requirements for Regulated Food and Feed Product Applications to Establish the Presence of Small Particles Including Nanoparticles. EFSA J..

[76] European Chemicals Agency (ECHA) (2022). Guidance on Information Requirements and Chemical Safety Assessment. Appendix R7-1 for Nanomaterials Applicable to Chapter R7a and R7c Endpoint Specific Guidance, Version 4.0.

[77] U.S. Food and Drug Administration (FDA) (2014). Considering Whether an FDA-Regulated Product Involves the Application of Nanotechnology: Guidance for Industry.

[78] Cheng Y., Yu Q., Zhang W., Liu Z., Ding J., Pan H., Li Y., Wu D., Zhu M., Xie X. (2025). Diet Dependent Trophic Transfer of Nanoparticles (ZnO and TiO_2_) along the “Photic Biofilm-Snail” Food Chain. J. Hazard. Mater..

[79] Xin X., Huang G., Zhang B., Zhou Y. (2021). Trophic Transfer Potential of nTiO_2_, nZnO, and Triclosan in an Algae-Algae Eating Fish Food Chain. Aquat. Toxicol..

[80] Chen X., Zhu Y., Yang K., Zhu L., Lin D. (2019). Nanoparticle TiO_2_ Size and Utile Content Impact Bioconcentration and Biomagnification from Algae to Daphnia. Environ. Pollut..

[81] Zhu X., Wang J., Zhang X., Chang Y., Chen Y. (2010). Trophic Transfer of TiO_2_ Nanoparticles from Daphnia to Zebrafish in a Simplified Freshwater Food Chain. Chemosphere.

